# Comparison of the Simulated Response of Three *in Silico* Human Stem Cell-Derived Cardiomyocytes Models and *in Vitro* Data Under 15 Drug Actions

**DOI:** 10.3389/fphar.2021.604713

**Published:** 2021-03-15

**Authors:** Michelangelo Paci, Jussi T. Koivumäki, Hua Rong Lu, David J. Gallacher, Elisa Passini, Blanca Rodriguez

**Affiliations:** ^1^ BioMediTech, Faculty of Medicine and Health Technology, Tampere University, Tampere, Finland; ^2^ Global Safety Pharmacology, Discovery Sciences, Janssen Research and Development, Janssen Pharmaceutica NV, Beerse, Belgium; ^3^ Department of Computer Science, University of Oxford, Oxford, United Kingdom

**Keywords:** human stem cell-derived cardiomyocyte, action potential, calcium transient, *in silico* modeling, drug test, sensitivity analysis

## Abstract

**Objectives:** Improvements in human stem cell-derived cardiomyocyte (hSC-CM) technology have promoted their use for drug testing and disease investigations. Several *in silico* hSC-CM models have been proposed to augment interpretation of experimental findings through simulations. This work aims to assess the response of three hSC-CM *in silico* models (Koivumäki2018, Kernik2019, and Paci2020) to simulated drug action, and compare simulation results against *in vitro* data for 15 drugs.

**Methods:** First, simulations were conducted considering 15 drugs, using a simple pore-block model and experimental data for seven ion channels. Similarities and differences were analyzed in the *in silico* responses of the three models to drugs, in terms of Ca^2+^ transient duration (CTD_90_) and occurrence of arrhythmic events. Then, the sensitivity of each model to different degrees of blockage of Na^+^ (I_Na_), L-type Ca^2+^ (I_CaL_), and rapid delayed rectifying K^+^ (I_Kr_) currents was quantified. Finally, we compared the drug-induced effects on CTD_90_ against the corresponding *in vitro* experiments.

**Results:** The observed CTD_90_ changes were overall consistent among the *in silico* models, all three showing changes of smaller magnitudes compared to the ones measured *in vitro*. For example, sparfloxacin 10 µM induced +42% CTD_90_ prolongation *in vitro*, and +17% (Koivumäki2018), +6% (Kernik2019), and +9% (Paci2020) *in silico*. Different arrhythmic events were observed following drug application, mainly for drugs affecting I_Kr_. Paci2020 and Kernik2019 showed only repolarization failure, while Koivumäki2018 also displayed early and delayed afterdepolarizations. The spontaneous activity was suppressed by Na^+^ blockers and by drugs with similar effects on I_CaL_ and I_Kr_ in Koivumäki2018 and Paci2020, while only by strong I_CaL_ blockers, e.g. nisoldipine, in Kernik2019. These results were confirmed by the sensitivity analysis.

**Conclusion:** To conclude, The CTD_90_ changes observed *in silico* are qualitatively consistent with our *in vitro* data, although our simulations show differences in drug responses across the hSC-CM models, which could stem from variability in the experimental data used in their construction.

## Introduction

Human stem cell-derived cardiomyocytes (hSC-CMs) have emerged as a promising tool for translational research, cardiac repair and drug development (Bezzerides et al., 2017; Miyagawa and Sawa, 2018; de Korte et al., 2020). After the initial hype, it has, however, become widely acknowledged that with current methods and technology hSC-CMs have a fundamental limitation: the cells cannot be fully matured into human ventricular cardiomyocytes (hV-CMs). Instead, hSC-CMs have a distinct phenotype of their own that shares features with partially differentiated developing cells (Khan et al., 2013). Furthermore, hSC-CMs show less robustness and greater tendency for arrhythmic events than native hV-CMs; a phenomenon that appears to be linked to a weaker repolarization reserve (Paci et al., 2015; Koivumäki et al., 2018; Lemoine et al., 2018; Treat et al., 2019). Thus, although hSC-CMs do resolve the problem of species-dependent differences related to use of animal models, the translation of data obtained from hSC-CMs to humans is not straightforward.

Computational modeling and simulation offer tools to augment experimental investigations by aiding their interpretation, and identifying possible sources and modulators of hSC-CM electrophysiology. For example, *in silico* approaches have already been successfully employed to investigate the translatability of drug responses from immature hSC-CMs to mature hV-CMs (Gong and Sobie, 2018; Tveito et al., 2018), creating a math-based and mechanistic virtual link to the phenotype of hV-CMs. *In silico* hSC-CM models also helped to gain insight on specific features of hSC-CMs, e.g. automaticity and its causes (Koivumäki et al., 2018; Paci et al., 2020), and to tackle the huge electrophysiological variability observed in action potentials (AP) and Ca^2+^ transients (CaT) recorded *in vitro* (Paci et al., 2017; Kernik et al., 2019), e.g. showing potential combination of ion currents that could make cardiac cells more prone to develop arrhythmic behaviors in response to drugs or in presence of disease phenotypes (Paci et al., 2017; Paci et al., 2018a; Kernik et al., 2020). Currently, there are three pedigrees of hSC-CM *in silico* models, all parametrized on hSC-CM data, developed and published by 1) Paci et al., 2) Koivumäki et al., and 2) Kernik et al. (Koivumäki et al., 2018; Kernik et al., 2019; Paci et al., 2020).

The goal of this work is to investigate differences and similarities in the response of the three latest hSC-CM models to 15 different drugs, and their comparison against an *in vitro* hSC-CM experimental dataset. We expect the insights from our study will enable other researchers to select the most appropriate model for their specific investigations.

## Methods

### Model Components and Structures

Table 1 shows the number of compartments and the ion currents, pumps and exchangers included in the three considered computational (*in silico*) cellular models: Kernik2019 (Kernik et al., 2019), Koivumäki2018 (Koivumäki et al., 2018), and Paci2020 (Paci et al., 2019; Paci et al., 2020). They are all single cell models, with a similar formulation for certain ionic currents, as they are all based on the original hSC-CM model from Paci et al. (Paci et al., 2013). The following currents/fluxes are modeled using the Hodgkin-Huxley paradigm, even if with different formulations: fast Na^+^ current (I_Na_), funny current (I_f_), L-type Ca^2+^ current (I_CaL_), rapid and slow delayed rectifier K^+^ currents (I_Kr_ and I_Ks_), inward rectifying K^+^ current (I_K1_), transient outward K^+^ current (I_to_). Of note, I_CaL_ driving force is formulated according to the Goldman-Hodgkin-Katz flux equation in Paci2020 and Kernik2019. Na^+^ and Ca^2+^ background currents were represented as resistive fluxes. The Na^+^/K^+^ pump (I_NaK_), the Na^+^/Ca^2+^ exchanger (I_NCX_) and the Ca^2+^ sarcolemmal pump (I_pCa_) follow the formulation used in (ten Tusscher et al., 2004). Kernik2019 and Koivumäki2018 use two different Markov formulations for the Ca^2+^ release from the sarcoplasmic reticulum (SR) (Shannon et al., 2004) and (Keizer and Levine, 1996), respectively. Conversely, Paci2020 uses a Hodgkin-Huxley formulation. The Kernik2019 model is the only one including the T-type Ca^2+^ current (I_CaT_), as well as the Na^+^ and K^+^ fluxes via I_CaL_ channels. Paci2020 is the only model including the late Na^+^ (I_NaL_) current (Ma et al., 2013). Koivumäki2018 is the only model including the inositol 1,4,5 triphosphate (IP3) receptor-mediated Ca^2+^ release (J_IP3_).

**TABLE 1:** Summary of the compartments and the ion currents and fluxes included in the three models.

	Paci2020	Koivumäki2018	Kernik2019
Compartments	2	61	2
I_Na_	HH	HH	HH
I_NaL_	HH	—	—
I_f_	HH	HH	HH
I_CaL_ Ca^2+^	HH, GHK	HH	HH, GHK
I_CaL_ Na^+^	—	—	HH, GHK
I_CaL_ K^+^	—	—	HH, GHK
I_CaT_	—	—	HH
I_Kr_	HH	HH	HH
I_Ks_	HH	HH	HH
I_K1_	HH	HH	HH
I_to_	HH	HH	HH
I_NCX_	TT	TT	TT
I_NaK_	TT	TT	TT
I_pCa_	TT	TT	TT
I_bNa_	HH, R	HH, R	HH, R
I_bCa_	HH, R	HH, R	HH, R
J_RyR_	HH, R	M^2^	M^1^
J_SERCA_	TT	TT	TT
J_leak_	HH, R	HH, R	HH, R
J_IP3_	—	M^3^	—

We reported the modeling paradigm for each ion current/flux: Hodgkin and Huxley (HH), Hodgkin and Huxley as resistive current/flux (HH,R), Hodgkin and Huxley gates with Goldman-Hodgkin-Katz driving force (HH,GHK), Markov (M), based on the TenTusscher2004 model (ten Tusscher et al., 2004) (TT).

^1^ (Shannon et al., 2004),

^2^ (Keizer and Levine, 1996),

^3^ (Sneyd and Dufour, 2002).

The three models show differences in their compartmental structure and description of Ca^2+^ dynamics. Paci2020 and Kernik2019 share the same cylindrical structure and compartmentalization: one compartment for cytosol and one for SR. Koivumäki2018 includes an additional Ca^2+^ outward flux from the SR, transmitted by IP3, and formulated as a Markov model. Moreover, Koivumäki2018 has a more complex layered “onion-like” compartmentalization for Ca^2+^. In order to describe the Ca^2+^ spatial distribution and diffusion, the CM is approximated as a sphere whose radius from SR towards the sarcolemma is divided in 61 sub-compartments. This enables a more refined description of the cytosolic Ca^2+^ dynamics, with no need for partial differential equations, at the price of longer computing time, as shown in the Results section.

### *In vitro* Drug Data

A total of 15 drugs were tested *in vitro* at multiple concentrations: antiarrhythmic drugs (bepridil, dofetilide, flecainide, lidocaine, mexiletine, procainamide and verapamil), an antiepileptic drug (phenytoin), antibiotics (moxifloxacin and sparfloxacin), hypertension drugs (nimodipine and nisoldipine), anticonvulsants (primidone), antianginals (ranolazine) and Ba^2+^ salts (BaCl_2_). Data were the same used in (Passini et al., 2017).

Briefly, CaT recordings from hSC-CMs (Cor.4U) were acquired at 37°C and 5% CO_2_ on pre-plated preparations from Axiogenesis (Cologne, Germany). Cells were seeded in fibronectin-coated 96-well plates at a density suited to form a monolayer and maintained in culture in a stage incubator. The tested 15 drugs were dissolved in DMSO to obtain a stock solution of 1,000x the highest test concentration. Further dilutions were made with DMSO to obtain concentrations of 1,000x intended concentration. On the experiment day these solutions were diluted with Tyrode (Sigma), supplemented with 10 mM HEPES to solutions containing twice the intended concentration (compound plate: 2x intended concentration). Final DMSO concentration in test solutions and vehicle control was 0.1%. Full method details are reported earlier in (Lu et al., 2019) and (Kopljar et al., 2018). Ca^2+^ transients (CaT) recorded as the calcium dye-fluorescence signal integrated over the whole well. CaT duration at 90% of the initial base value (CTD_90_) was quantified, as it is known to be correlated with APD (Gauthier et al., 2012; Spencer et al., 2014), similarly to other studies (Lu et al., 2015; Zeng et al., 2016). The CTD_90_ values used in this study were measured from 24 to 28 min after compound addition. Therefore, an excessive increment of CTD_90_, as well as an anomalous CaT time-course, can be considered as markers of APD or QT prolongation and pro-arrhythmic risk - early afterdepolarization (EAD) or torsades de pointes (TdP).

### *In silico* Drug Trials

All the drugs and concentrations tested *in vitro* were simulated *in silico* using the three hSC-CM models. Drug effects on seven ion currents (I_Na_, I_Kr_, I_CaL_, I_NaL_, I_Ks_, I_to_ and I_K1_) were simulated using the single pore block model (Brennan et al., 2009; Paci et al., 2015; Williams and Mirams, 2015), formulated as

$${\text{I}}_{i}\left(\left[D\right]\right)=\frac{{\text{I}}_{i}^{\prime }}{1+{\left(\left[D\right]/{\text{IC}}_{50}\right)}^{n_{H}}},$$

where I_i_ is the ion current corresponding to the drug concentration [D], I_*i*_′ the ion current in control conditions, IC_50_ is the half-maximal dose response and n_H_ is the Hill coefficient. The used IC_50_ and Hill coefficients are reported in Supplementary Table S1 (Passini et al., 2017). For seven drugs, multiple (IC_50_, Hill coefficient) pairs were considered, as in (Passini et al., 2017). Each model was run for 800 s to reach the steady state. For each drug, concentration and model, simulations were run for 200 s starting from steady state, and then CTD_90_ was computed on the last eight spontaneous CaTs, and the occurrence of proarrhythmic events was assessed. No external pacing was applied to the three models. Since each of the models was tuned and validated on a specific working point, we used the baseline models as originally published by the authors.

### Arrhythmic Event Classification

Abnormal rhythms *in vitro* were visually assessed from CaT traces. *In silico*, they were defined based on the AP time-course, as in (Paci et al., 2020). Five types of arrhythmic events were considered. Early after-depolarizations (EADs) were defined as aberrant and transient reversals of the membrane potential repolarization (during phase two or three of the AP) or of the CaT decay. Delayed afterdepolarizations (DADs, observed only *in silico*) were defined as transient depolarizations of the membrane potential or increments of the cytosolic Ca^2+^ from its basal value during their diastolic phases. EADs and DADs were visually assessed. Repolarization failure (RF), i.e. when the membrane potential or the cytosolic Ca^2+^ do not return to their diastolic values for several seconds, was identified when a stable (maximum upstroke velocity smaller than 0.2 V/s) membrane potential >−40 mV was observed during the last 15 s of simulation. Irregular rhythm (IRR) was identified if the difference in cycle length between two consecutive APs or CaTs was greater than 150%. Spontaneous rate greater than 2 Hz was labeled as ventricular tachycardia-like rhythm (VT, observed only *in vitro*).

We also identified two patterns related to the cessation of the spontaneous electrical activity of hSC-CMs, that we did not considered pro-arrhythmic: quiescence (Q) and residual activity (RESAC). If the membrane potential showed small peaks, greater than −40 mV but smaller than 0 mV, not developing APs, the model was labeled as RESAC. Conversely, we considered the model quiescent (Q), i.e. not producing spontaneous AP and CaTs, if during the last 15 s the average membrane potential was smaller than −40 mV or the residual activity’s peak potentials were all the smaller than −40 mV.

### Sensitivity to Simultaneous Current Blocks

A systematic sensitivity analysis was also conducted by considering simultaneous blocks of I_Kr_ vs I_CaL_ and I_Na_ vs I_CaL_ by blocking (0, 20, 40, 60, 80, 100)% of each current (thirty six tests for each couple of currents). For each test we assessed if the model 1) produced spontaneous APs, 2) triggered repolarization abnormalities or irregular rhythms, 3) showed quiescence or residual activity without or 4) with irregular rhythm after drug administration. Simulations with Paci2020 and Kernik2019 models were run for 500 s, while simulations with Koivumäki2018 were run for 200 s only, due to the long computing time required by this model (only the tests showing irregular rhythm were extended until 500 s to check if it persisted or turned into quiescence or residual activity).

## Results

### Simulations With the Three Models Yield Different Action Potentials and Ca^2+^ Transients Shapes

**FIGURE 1:**
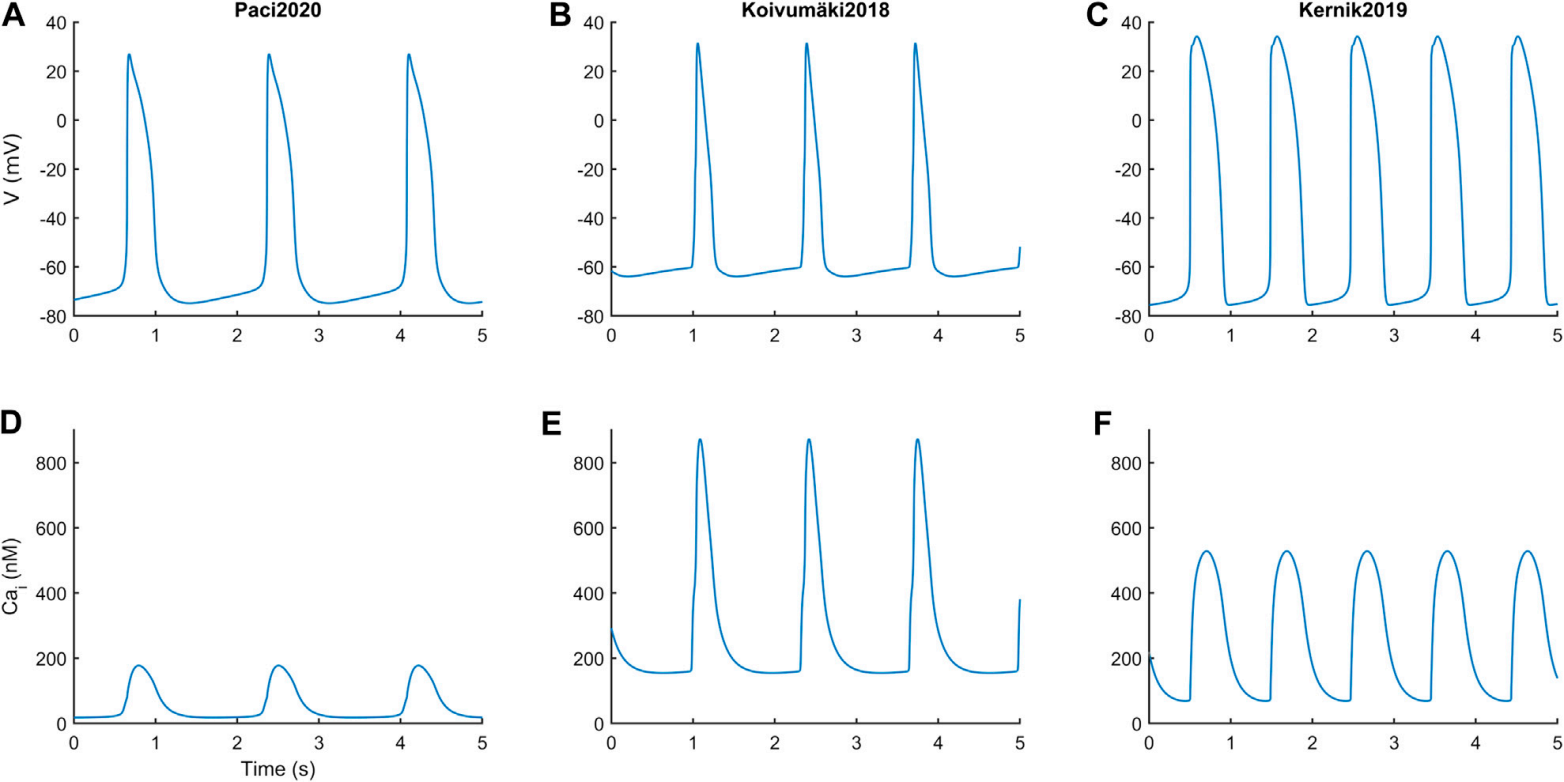
Action potentials **(A and B,** **C)** and Ca^2+^ transients **(D and E, F)** in steady state for the three *in silico* hSC-CM models.

Figure 1 reports the spontaneous APs and CaTs produced by the three models in steady state, and Table 2 summarizes the main AP and CaT morphological features. The AP and CaT traces are quite different, reflecting the fact that hSC-CMs can exhibit remarkable differences, *in silico* as well as *in vitro*, e.g. different AP morphologies (Paci et al., 2017; Kernik et al., 2019). In terms of APs, Koivumäki2018 shows the most triangular ones, with depolarized maximum diastolic potential (MDP) and the shortest AP duration at 90% (APD_90_). Paci2020 and Kernik2019 show quite similar AP features, in agreement with (Ma et al., 2011), except for the rate of the spontaneous APs, which is almost double in the latter.

**TABLE 2:** Morphological features from spontaneous action potentials (APs) and Ca^2+^ transients (CaTs).

Morphological feature	Paci2020	Kernik2019	Koivumäki2018
APA (mV)	102	110	95
MDP (mV)	−75	−76	−64
APD_90_ (ms)	390	414	247
AP_Tri (-)	2.8	2.6	1.7
Rate (bpm)	35	61	45
Diastolic Cai (nM)	18	68	154
Systolic Cai (nM)	177	529	873
CTD_90_ (ms)	550	601	407

APA: AP amplitude. MDP: maximum diastolic potential. APD_90_: AP duration at 90% of repolarization. AP_Tri: AP triangulation ratio, defined as (APD_40_-APD_30_)/(APD_80_-APD_70_). Rate: rate of the spontaneous APs/CaTs. CTD_90_: CaT duration at 90% decay.

In terms of CaT, Paci2020 shows the lowest diastolic and systolic cytosolic Ca^2+^ levels, and CaT amplitude is in agreement with the measurements by Rast et al. (Rast et al., 2015). The Kernik2019 CaT was calibrated on the data from Garg et al. (Garg et al., 2018), after their conversion from fluorescence level to actual concentrations.

**FIGURE 2:**
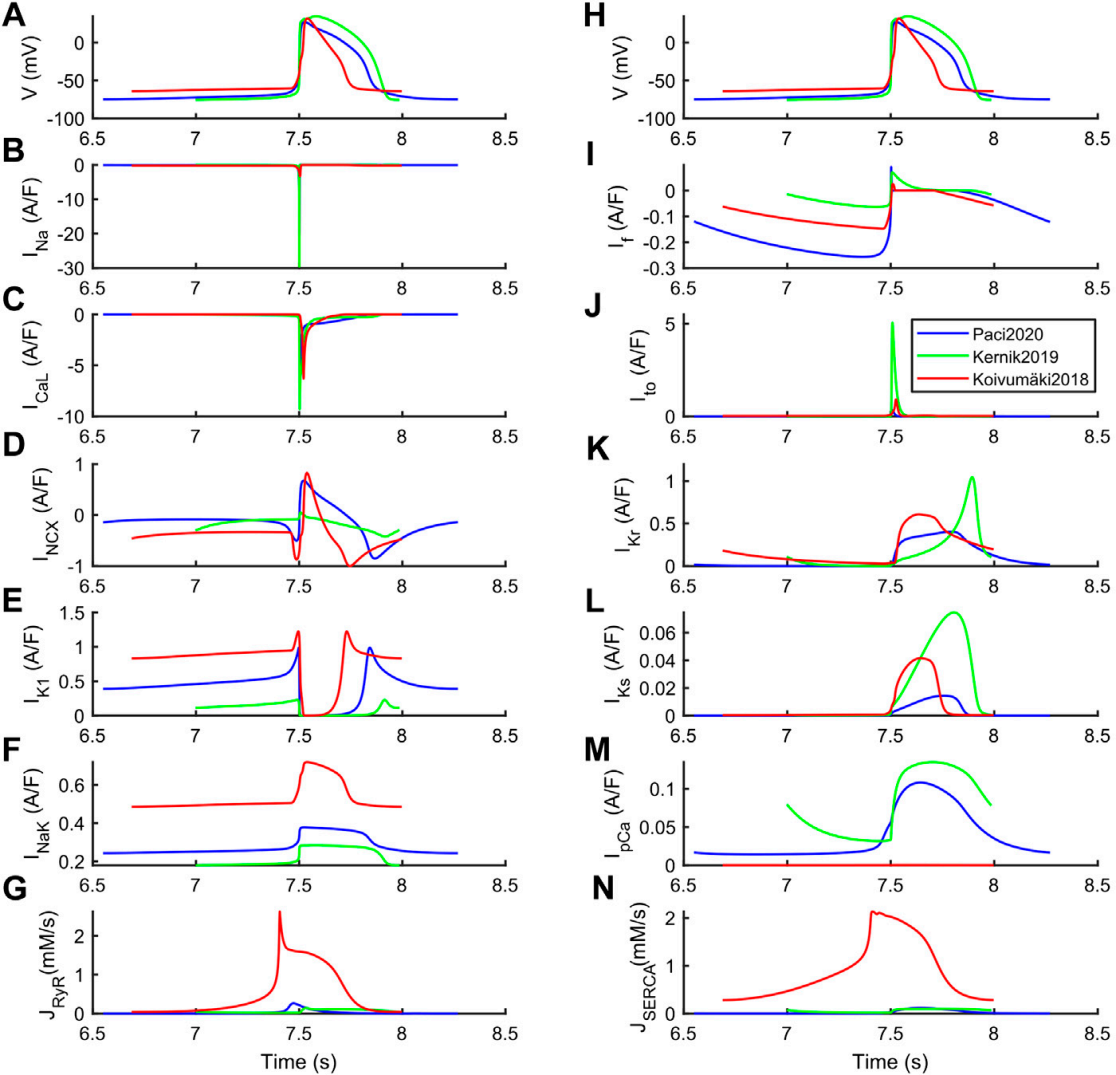
Ion currents underlying the steady-state action potentials in the Paci2020 (blue), Kernik2019 (green) and Koivumäki2018 (red) models. **(A and H)** Membrane potential. **(B)** Fast Na^+^ current (I_Na_). **(C)** L-type Ca^2+^ current (I_CaL_). **(D)** Na^+^-Ca^2+^ exchanger (I_NCX_). **(E)** Inward rectifying K^+^ current (I_K1_). **(F)** Na^+^-K^+^ pump (I_NaK_). **(G)** Ca^2+^ release from sarcoplasmic reticulum (J_RyR_). **(I)** Funny current (I_f_). **(J)** Transient outward K^+^ current (I_to_). **(K)** Rapid delayed rectifying K^+^ current (I_Kr_). **(L)** Slow delayed rectifying K^+^ current (I_Ks_). **(M)** Sarcolemma Ca^2+^ pump (I_pCa_). **(N)** SERCA pump (J_SERCA_).

The differences among the three models can be explained based on their steady state ion currents and fluxes, reported in Figure 2 and further expanded in Supplementary Figures S1–S3. Kernik2019 has the greatest I_Na_, I_CaL_, I_to_, I_Kr_ and I_Ks_. Conversely, it has the smallest I_K1_, and the highest rate of spontaneous APs (a 20% I_K1_ increment would reduce the spontaneous rate to 38 bpm, similar to the other two models). Paci2020 and Koivumäki2018 show a very similar I_NCX_ shape, coming from the fact that in both models the automaticity is sustained by the pre-upstroke inward component of I_NCX_ (only partly for Paci2020). This pre-upstroke I_NCX_ inward component is missing in Kernik2019. Paci2020 and Koivumäki2018 also have a similar I_CaL_ peak current, and Koivumäki2018 shows the smallest I_Na_ across the three models.

These differences are particularly relevant to understand the different responses to some of the drugs, reported in the next section. The structural differences in the three models are also reflected in their running time. Being characterized by simpler structure and compartmentalization, Paci2020 and Kernik2019 are faster (210 s simulations take 22 s). Conversely, due to its higher complexity, Koivumäki2018 is more than 100 times slower (210 s simulations take 2,331 s). These benchmark simulations were run in Matlab 2017b on a laptop computer (i7 @2.80 GHz and 32 GB memory).

### *In silico* drug tests: characterization of diverse responses in the three models

**FIGURE 3:**
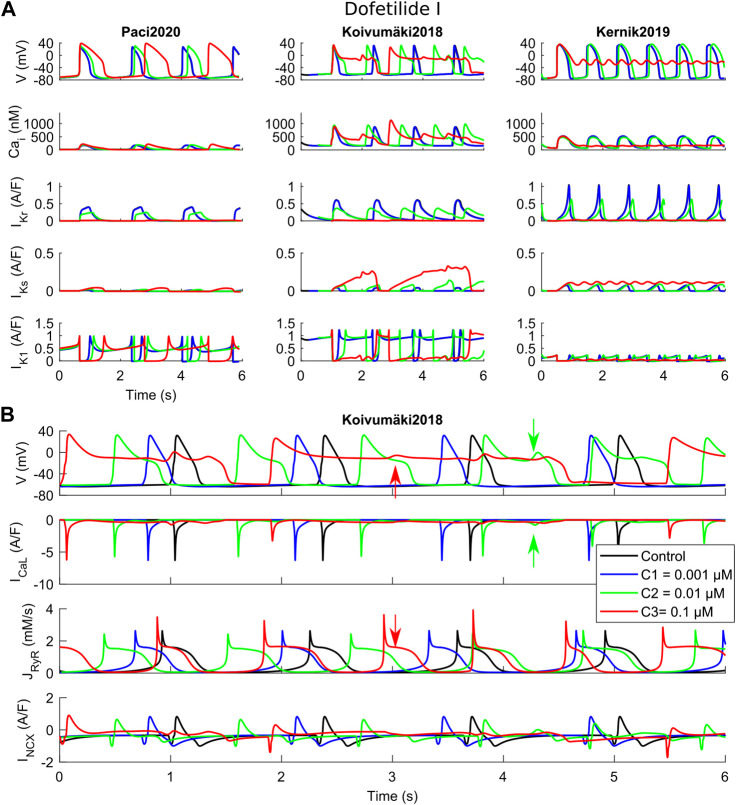
Effects of dofetilide I on the three in silico models for the three tested concentrations (C1, C2 and C3). **(A)** Comparison of action potentials, Ca^2+^ transients and the main K^+^ currents. **(B)** Detail of the Koivumäki2018 traces, showing the development of EADs following reactivation of I_CaL_ (green arrows) and spontaneous Ca^2+^ releases from the sarcoplasmic reticulum (red arrows).

**FIGURE 4:**
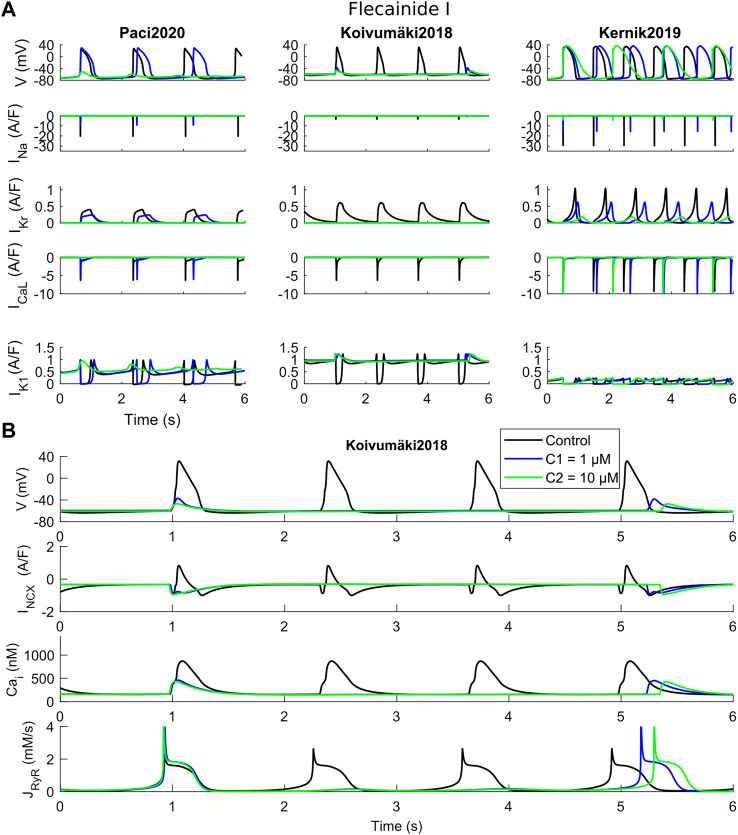
Effects of flecainide I on the three in silico models for the two tested concentrations (C1 and C2). **(A)** Comparison of action potentials, Ca^2+^ transients and ion currents. **(B)** Detail of the Koivumäki2018 traces, showing a residual activity due to spontaneous Ca^2+^ releases from the sarcoplasmic reticulum that activate the inward component of I_NCX_.

**FIGURE 5:**
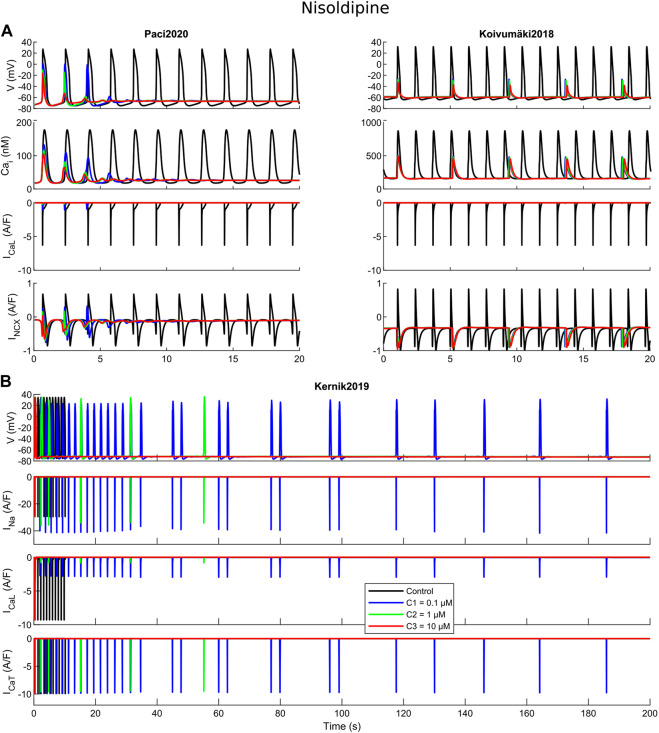
Cessation of the spontaneous electrical activity upon administration of nisoldipine. For all the three tested concentrations (C1, C2 and C3), Paci2020 becomes quiescent and Koivumäki2018 maintains a residual electrical activity not sufficient to trigger action potentials. For concentrations one and two, Kernik2019 shows an irregular rhythm before quiescence, while concentration three terminates immediately the automaticity.

Before proceeding with an *in vitro*–*in silico* comparison, we aim to show in detail how the three models react to a specific set of three drugs, namely dofetilide (Figure 3), flecainide (Figure 4) and nisoldipine (Figure 5), each one highlighting a specific mechanism of action.

Dofetilide (Figure 3) is a well-known hERG blocker that triggers AP prolongation and repolarization abnormalities, such as EAD and RF. The rationale behind this test is to show how these phenomena are recapitulated by the three models in response to a strong hERG block. These phenomena are recapitulated, as shown in Figure 3A, when considering dofetilide I IC_50_ as in Supplementary Table S1. For the two lowest doses, 0.001 and 0.01 µM, dofetilide prolongs the APs and CaTs in all three models. However, at the highest concentration (0.1 µM, corresponding to an I_Kr_ 96% block), dofetilide triggers three different behaviors. It prolongs the AP and CaT durations in Paci2020, but it does not trigger any repolarization abnormality. In this model, I_Ks_ and the strong I_K1_ can support the late repolarization phase, despite the strong AP prolongation. Conversely, Kernik2019 has a small I_K1_ compared to Paci2020 and Koivumäki2018, which makes it more difficult to stabilize the membrane potential to its diastolic value. In fact, at the highest dofetilide concentration, Kernik2019 fails to repolarize in the late repolarization phase and it sets around −20 mV. Particular attention is paid to Koivumäki2018, which triggers EADs at both intermediate and high concentrations, showing two different mechanisms. At the intermediate concentration, I_CaL_ reactivation occurs, due to the prolonged AP (Figure 3B, green arrows). This influx of positive charges depolarizes the membrane potential and generates the EAD. In this example, all the Ca^2+^ releases from the SR are synchronized with the AP upstrokes. At the highest concentration, spontaneous releases of Ca^2+^ from the SR occur (Figure 3B, red arrows). They are not synchronized with the AP upstrokes, and instead they happen during the prolonged repolarization phase. Such releases induce an increment in the cytosolic Ca^2+^ concentration, which enhances the inward I_NCX_ component, and then triggers the EAD.

Flecainide blocks primarily I_Kr_ and I_Na_, but also I_CaL_ to a minor extent, as shown in Supplementary Table S1. Figure 4 shows APs and CaTs when considering flecainide I (as defined in Supplementary Table S1). The lowest concentration (1 µM) blocks I_Na_, I_Kr_ and I_CaL_ by 30%, 40% and 4%, while the intermediate concentration (10 µM) blocks the same currents by 69%, 81% and 28%, respectively. The results illustrate how flecainide stops spontaneous electrical activity, or allows only a residual activity, in Paci2020 and Koivumäki2018, whereas with the Kernik2019 model, beating still occurs spontaneously, showing stronger automaticity. Indeed, in agreement with our *in vitro* data (Supplementary Figure S7), both Paci2020 and Kernik2019 show AP/CaT prolongation at low dose. However, with Paci2020 spontaneous activity stops, not sustained anymore by I_Na_ which is blocked by 69%, while Kernik2019 still produces APs. This phenomenon is even clearer in Koivumäki2018, where I_Na_ is extremely small also in control conditions. Already at the low concentration, APs do not develop anymore, but there is a residual activity, only due to spontaneous Ca^2+^ releases from SR (about every 4 s). These releases trigger the inward I_NCX_ component that slightly depolarizes the membrane potential. Conversely, Kernik2019 maintains its automaticity, despite the strong I_Na_ block: indeed, the authors of the Kernik2019 model reported that the model spontaneously beats also in case of full I_Na_ block. However, flecainide at the intermediate concentration does induce a prolongation of APs and CaTs in the Kernik2019 model.

Nisoldipine is a powerful I_CaL_ blocker, with minor effects also on I_Na_, I_NaL_, I_Kr_ and I_Ks_. The three concentrations considered here, 0.1, 1 and 10 μM, induced I_CaL_ blocks of 85%, 97% and 99%. The rationale behind this test is showing how a strong I_CaL_ blocker can affect the automaticity in the three models. Few seconds after drug administration, Paci2020 and Koivumäki2018 show complete quiescence and a residual activity, respectively, at all three nisoldipine concentrations. The importance of I_CaL_ in the first two models is related to the role of Ca^2+^ handling and I_NCX_ in sustaining the automaticity. As reported in (Koivumäki et al., 2018; Paci et al., 2020), one mechanism sustaining the automaticity is a small inward component of I_NCX_ inducing the depolarization of the membrane potential until it reaches the activation threshold for I_Na_. After I_CaL_ block, less Ca^2+^ enters the cytosol and is available for the Ca^2+^-induced Ca^2+^ release, and the Ca^2+^ transient pre-upstroke component becomes smaller and smaller and this reduces the activity of I_NCX_ that cannot support anymore proper automaticity. This phenomenon is particularly clear in the Paci2020 model (Figure 5A). Koivumäki2018 shows a residual activity since it has a greater spontaneous Ca^2+^ release from SR (Figure 2) that can still trigger the membrane potential depolarization via I_NCX_ up to about -40 or -30 mV, but not enough to trigger a full AP. I_CaL_ is also fundamental in Kernik2019. Actually, spontaneous APs in Kernik2019 rely more on I_CaL_ than I_Na_, even in their upstroke phase. This model continues to beat spontaneously with I_Na_ full block (tested up to 1,000 s, not shown): I_CaL_ can compensate the lack of I_Na_ and sustain the upstroke. On the other hand, when I_CaL_ is blocked (e.g. by concentration one), the spontaneous activity slows down and becomes irregular, until it stops. Kernik2019 can also count on an additional depolarizing current, not present in the other two models: I_CaT_. Combining nisoldpine at concentration two with the full I_CaT_ block, the automaticity terminates upon *in silico* drug administration.

**FIGURE 6:**
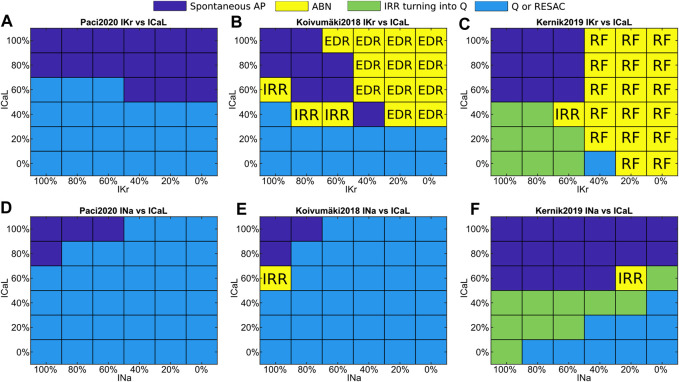
Sensitivity maps to simultaneous I_Kr_ and I_CaL_ blocks **(A and B, C)** or I_Na_ and I_CaL_ blocks **(D and E, F)**. Color code: Spontaneous APs, blue; quiescence or residual activity, cyan; transient irregular rhythm turning into quiescence, green; arrhythmic events, yellow. The yellow squares include also the event type: irregular rhythm (IRR), repolarization failure (RF), and simultaneous occurrence of EAD, DAD and repolarization failure (EDR).

In order to generalize the results obtained for dofetilide, flecainide and nisoldipine, we ran a sensitivity analysis by testing the simultaneous current blocks showed in Figure 6: I_Kr_ vs I_CaL_ (Figures 6A–C) and I_Na_ vs I_CaL_ (Figures 6D–F). Firstly, the Paci2020 model does not develop repolarization abnormalities in response to I_Kr_ block, Kernik2019 responds to high I_Kr_ block only with repolarization failures, while Koivumäki2018 develops multiple repolarization abnormalities (EADs, DADs and RF). Secondly, Koivumäki2018 and Paci2020 are more sensitive to I_Na_ block than Kernik2019 (Figures 6D–F). They show much smaller I_Na_ peaks (Figure 2), and this I_Na_ cannot sustain the upstroke in case of ∼50% block for Paci2020 and ∼30% block for Koivumäki2018. This is clear in case of blockade of Na^+^ currents (e.g., mexiletine I, II, III at concentration three and lidocaine II at concentration two), which impairs automaticity in all models but Kernik2019 (that shows spontaneous APs also with I_Na_ fully blocked) (Figure 6F). Thirdly, Koivumäki2018 and Paci2020 are the most sensitive to I_CaL_ block. Finally, Kernik2019 has the strongest automaticity. This has been confirmed also by additional tests (not shown), e.g. Kernik2019 spontaneous activity lasts about 400 s in conditions of 90% I_f_ block, and a 90% I_NCX_ block does not stop the spontaneous APs and CaTs as observed in Paci2020 and Koivumäki2018. For some of the drugs blocking at the same time I_CaL_ and I_Kr_ (e.g. moxifloxacin I, II at concentration two or bepridil I, II, III at concentration two), this enables Kernik2019 to trigger repolarization abnormalities typical of I_Kr_ block, when the other models show residual or cessation of spontaneous activity.

### *In silico* Drug Tests: Comparison With the *in vitro* Results

**FIGURE 7:**
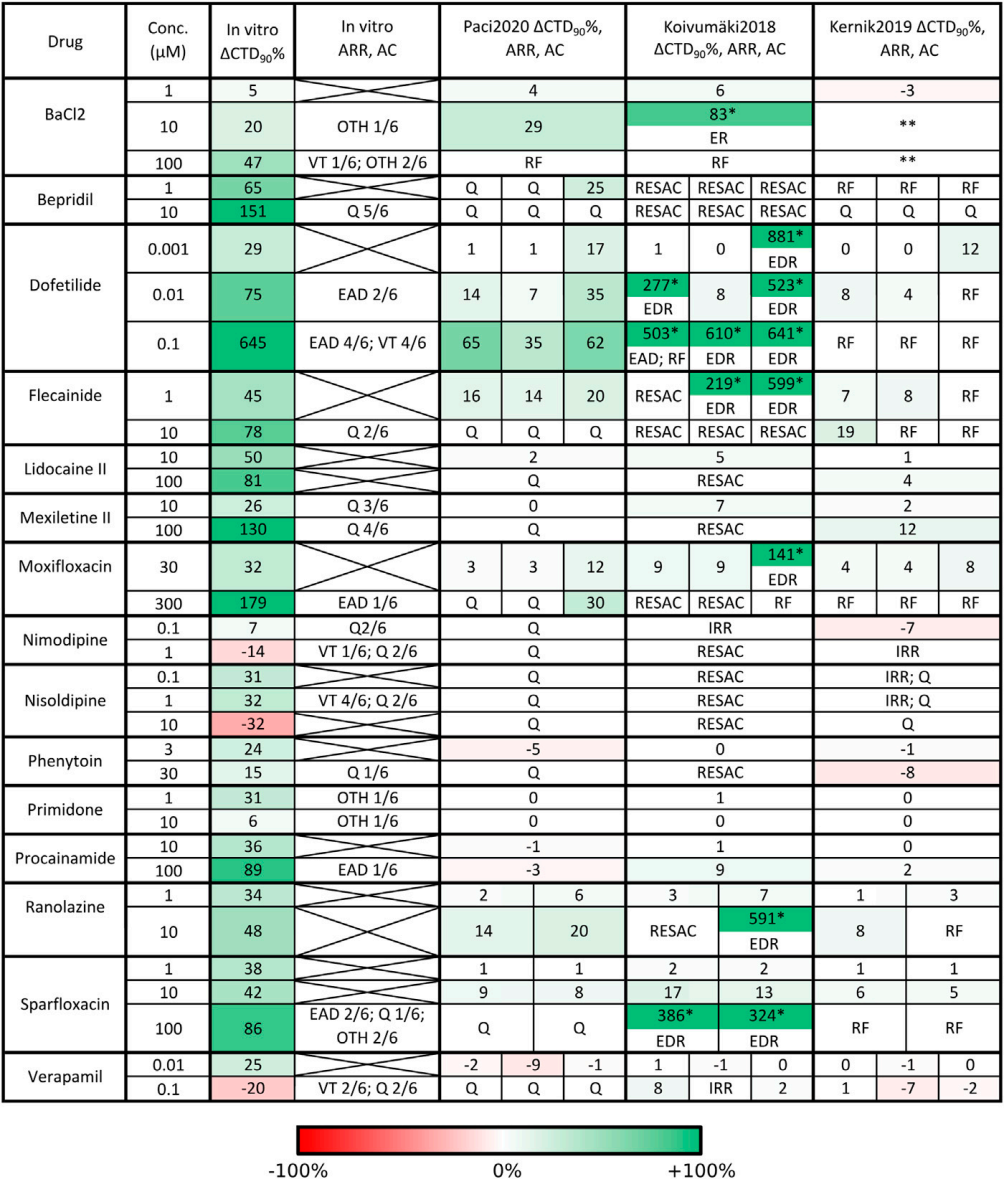
Qualitative and quantitative comparison of *in silico* drug tests in the three hSC-CM models against our *in vitro* data, for the 15 reference compounds. For each drug we report i) the tested concentrations, ii) the percent CaT duration variation with respect to baseline (ΔCTD_90_%), arrhythmic events (ARR) and automaticity cessation (AC) recorded *in vitro* and *in silico*. Arrhythmic events: early and delayed after-depolarization (EAD, DAD), repolarization failure (RF), irregular rhythm (IRR), ventricular tachycardia-like rhythm (VT) and other arrhythmic events (OTH). Automaticity cessation (AC): quiescence (Q) and residual activity (RESAC). Q and RESAC are not considered pro-arrhythmic. For Koivumäki2018, the ΔCTD_90_% marked with a star were computed on EADs and the model showed arrhythmic patterns including the combination of EADs, DADs and RF (shortened as EDR) or EADs and RF (shortened as ER). For Kernik2019, two stars in BaCl_2_ mean high rate Ca2^+^ oscillations and depolarized maximum diastolic potential, but no RF. When multiple combinations of IC_50_ and Hill coefficient were tested in simulation for the same compound, the corresponding *in silico* results consist of multiple sub-columns. In case of no *in vitro* arrhythmic events, the corresponding cell contains an X. An expanded version of this table is presented in the Supplementary material as Supplementary Table S2.

A detailed *in vitro*–*in silico* comparison is presented in Figure 7, Supplementary Figures S5–S7 and Supplementary Table S2. Figure 7 and Supplementary Table S2 report the *in silico* drug trial results and the comparison with the *in vitro* experiments in terms of CTD_90_ percent variations, occurrence of arrhythmic events, and cessation of automaticity, also for the alternative drug formulations not shown in Supplementary Figures S5–S7. In these three supplementary figures, we graphically report the percent *in vitro* and *in silico* CTD_90_ variations, arrhythmic events or the cessation of the spontaneous activity, and the main mechanisms of action, for the 15 drugs in the panel. If an *in vitro* or *in silico* CTD_90_ data is missing, it means that the spontaneous activity stopped, or an arrhythmic event prevented measuring CTD_90_. *In vitro*, most of the drugs induced prolongation of CTD_90_, except nimodipine, nisoldipine and verapamil. Bepridil, flecainide, lidocaine, mexiletine, phenytoin and ranolazine did not induce arrhythmic events, despite all but ranolazine stopping the spontaneous activity at the highest doses in at least a few cells.

The *in silico* results with the three models are in agreement (all showing consistent CTD_90_ prolongation or shortening). In absence of arrhythmic events, all the three models showed smaller CTD_90_ changes than *in vitro* experiments, e.g. sparfloxacin at concentration two prolonged by 42% *in vitro* CaTs, while *in silico* sparfloxacin I induced 17% prolongation in Koivumäki2018 and less than 10% prolongation in Paci2020 and Kernik2019. The extreme prolongations for Koivumäki2018 for dofetilide, flecainide II and III (concentration one), and moxifloxacin III (concentration one) are due to computing CTD_90_ manually on EADs. We did not compute the CTD_90_ for BaCl_2_ concentrations two and three for Kernik2019. In fact, I_K1_ block slightly depolarizes the maximum diastolic potential in Kernik2019 but not to the extent to trigger a repolarization failure (Supplementary Figure S4). However, the membrane potential and cytosolic Ca^2+^ start oscillating at a high rate and these oscillations do not have the morphology of CaTs.

**FIGURE 8:**
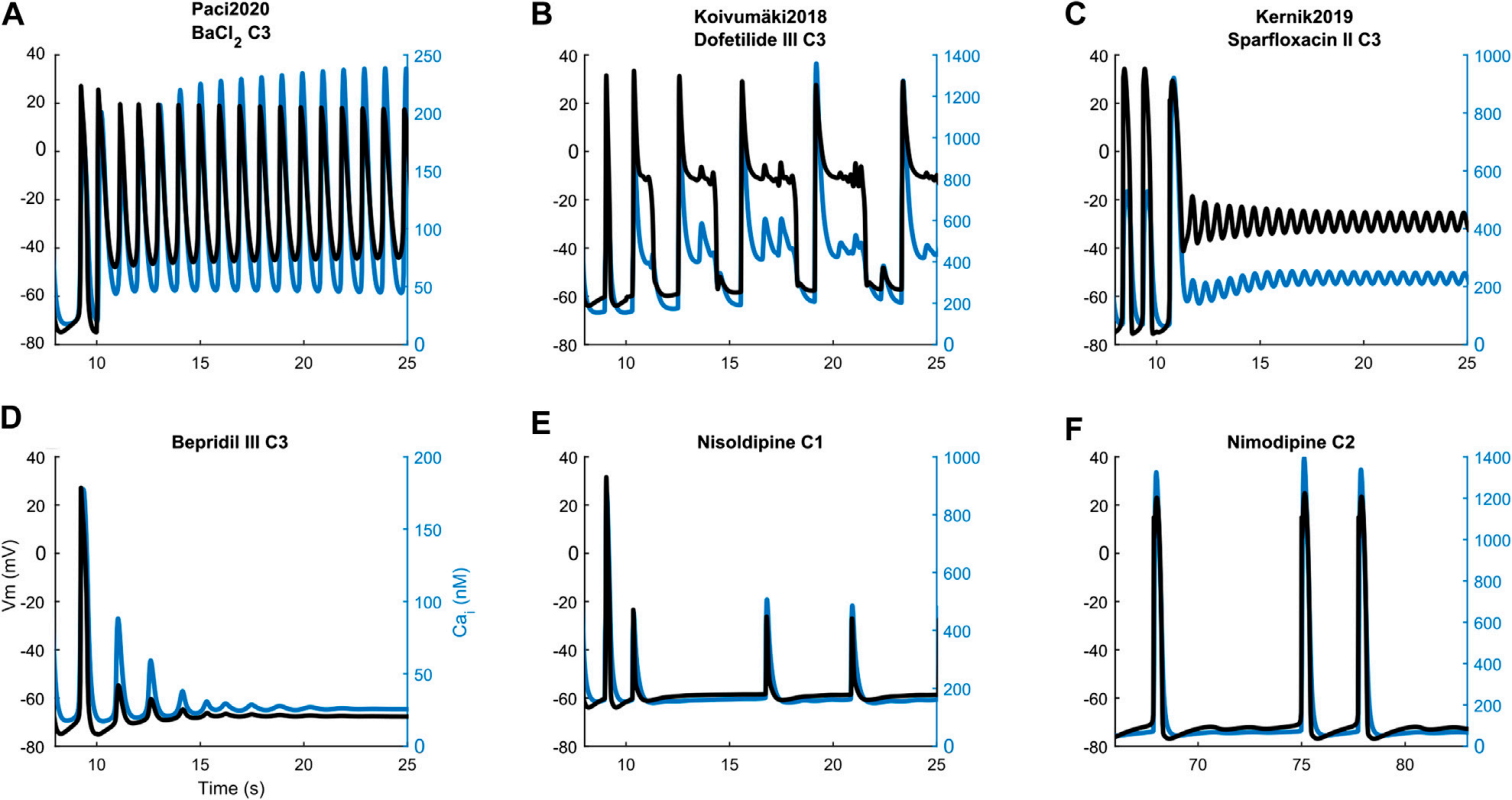
Illustrative action potential (black) and Ca^2+^ transient (blue) arrhythmic rhythms, abnormalities and the cessation of spontaneous activity produced by the three models. Paci2020: repolarization failure **(A)**, quiescence **(D)**. Koivumäki2018: early after-depolarizations **(B)** and residual activity **(E)**. Kernik2019: repolarization failure **(C)** and irregular rhythm **(F)**. The drug concentrations are reported as C1, C2 and C3, consistently with Supplementary Table S2.

Illustrative arrhythmic events and abnormal rhythms are presented in Figure 8. Paci2020 did not show arrhythmic events for any drug but for the maximum concentration of BaCl_2_ where it failed to repolarize (Figure 8A). It also showed the greatest CTD_90_ prolongations with no EADs among the three models (e.g. dofetilide III +62%). Furthermore, it simulated well the cessation of the spontaneous activity (Q) for drugs mainly blocking Na^+^ currents (e.g., mexiletine and lidocaine), I_CaL_ (e.g., nimodipine, nisoldipine and phenytoin), or a mixed combination of IC_50_ (e.g. bepridil, flecainide and sparfloxacin) (Figure 8D).

Conversely, Koivumäki2018 was the most sensitive one, triggering arrhythmic events, e.g. EAD and RF, for most of the drugs affecting the K^+^ currents (BaCl_2_, dofetilide and sparfloxacin) (Figure 8B). It never showed a complete stop of the spontaneous activity, but responded with a residual activity (RESAC), i.e. small depolarization of the membrane potential with peaks reaching about −30 mV, that cannot be considered APs (Figure 8E). This happened consistently with the quiescence of Paci2020 and the *in vitro* data, in case of drugs affecting mainly I_Na_ or I_CaL_.

Kernik2019 never produced EADs, but it responded with RF in most of the cases when the Koivumäki2018 model showed arrhythmogenic events (Figure 8C). Kernik2019 maintained also its automaticity in all the cases the Paci2020 and the Koivumäki2018 models showed quiescence or residual activity, except for nimodipine (Figure 8F) and nisoldipine, where it also showed cases of irregular rhythm (IRR). Nonetheless, these irregular rhythms lasted only 320 s (nimodipine concentration one) and 250 s (nisoldipine concentration one), then the automaticity stopped consistently with the other two models. None of the models showed ventricular tachycardia-like events that were on the contrary observed *in vitro*.

### *In silico* Drug Tests: Influence of Different IC_50_ and Ion Channel Characterizations

The same drug can have different effects in the same model (Figure 7), according to the drug characterization in terms of IC_50_ magnitude and how many ion channels were considered in the specific characterization. In fact, we used multiple characterizations for seven drugs, as in (Passini et al., 2017). For example, moxifloxacin I and II show consistent results in each of the three models. On the contrary, moxifloxacin III triggers different responses in Paci2020 (concentration two does not stop automaticity as it happens for moxifloxacin I and II) and Koivumäki2018 (concentration one triggers EADs and DADs instead of CTD_90_ prolongation and concentration two fails the repolarization). This is explained by the fact that characterizations moxifloxacin I and II show very similar IC_50_ and act on the same ion currents (I_Na_, I_Kr_ and I_CaL_), while moxifloxacin III does not block I_Na_ and I_CaL_ (that the sensitivity analysis demonstrated fundamental to keep the automaticity in Paci 2019 and Koivumäki2018), acting only on I_Kr_, I_Ks_ and I_NaL_ (which is present only in Paci2020 as individual ion current). Therefore, the results for moxifloxacin I and II are typical of an I_Na_ and/or I_CaL_ blocker, while moxifloxacin III behaves mainly as K^+^ current blocker. The same considerations can be also done for ranolazine I and II, which have different effects at concentration two in Koivumäki2018 (residual activity vs EADs and DADs).

## Discussion

In this work, we analyze and compare the simulated response of three recent *in silico* models of hSC-CM in control and under 15 drugs action, in terms of CaT duration, pro-arrhythmic behavior and automaticity. A comparison is also conducted between simulations with the three models and corresponding *in vitro* experiments on hSC-CMs. The generated insights provide a characterization and comparison of the three models that could inform selecting the model best suited for specific applications. The main contributions of our work are:

• The three models show qualitatively consistent *in silico* results in terms of CTD_90_ prolongation and shortening for specific drugs.
• The three models show different susceptibility to drug-induced pro-arrhythmic events. Paci2020 is the least prone to develop them, whereas Kernik2019 produces repolarization failures and Koivumäki2018 exhibits the highest variety of events (including EADs and DADs) and triggering mechanisms (I_CaL_ reactivation and spontaneous Ca^2+^ releases from SR).
• The strength of the automaticity is different across the three models. Kernik2019 shows the strongest spontaneous activity, supported by a combination of inward currents where I_CaL_ plays a fundamental role. Paci2020 and Koivumäki2018 are more sensitive to drug-induced impairment of automaticity, being mainly supported by Ca^2+^ handling and I_NCX_.
• The three models simulate CTD_90_ percent variations qualitatively in agreement, even though smaller, than the specific *in vitro* data considered here.
• For compounds with multiple experimental ion channel data, the sets of ion channels taken into account (e.g. I_Na_, I_Kr_, I_CaL_ vs I_Kr_ only) and the variations in IC_50_ and Hill values can lead to divergent simulation results, e.g. the AP/CaT prolongation and the occurrence of pro-arrhythmic events (typical of K^+^ current blockers), or the depolarization impairment and suppression of hSC-CM automaticity (typical of Na^+^ and Ca^2+^ blockers). The input ion channel data are therefore essential in determining the results of *in silico* drug tests.

### Origins and Basic Features of the Three Human Stem Cell-Derived Cardiomyocyte Models

Since the proposal of the Comprehensive *in vitro* Proarrhythmia Assay (CiPA) initiative (Colatsky et al., 2016; Strauss et al., 2019), the acceptance of hSC-CMs and *in silico* models in the field of pharmacology and drug safety has increased steadily. As a consequence, the number of hSC-CM *in silico* models available has also increased. At the time of the publication of the first *in silico* hSC-CM model (Paci et al., 2013), a limited amount of *in vitro* data was available in spite of the seminal work by Ma et al. (Ma et al., 2011), that represented the most complete characterization of ion currents and APs from control hSC-CMs. Due to the growing interest in the hSC-CM technology and its applications, additional experimental data were published, enabling the development of more sophisticated hSC-CM models (Paci et al., 2018b; Paci et al., 2020; Koivumäki et al., 2018; Kernik et al., 2019).

The first Paci model, Paci 2013 (Paci et al., 2013), was developed entirely based on the Ma et al. (Ma et al., 2011) dataset (Ma 2011) and additional *in vitro* data from cardiomyocytes derived from human embryonic stem cells for the ion currents missing in the Ma et al. dataset. Its structure closely resembles the structure of the TenTusscher hV-CM model (ten Tusscher et al., 2004). Over the years, many new Paci models were released (Paci et al., 2015; Paci et al., 2017; Paci et al., 2018b; Paci et al., 2020), integrating more mechanisms based on new *in vitro* data, e.g. I_NaL_ (Ma et al., 2013) or a more accurate Ca^2+^ handling calibrated on in-house measurements, described in (Paci et al., 2018b). The Paci2020 model used in this paper is the latest one and, in addition to being able simulate all the mechanisms successfully reproduced by its predecessors, it also encapsulates a more physiological description of the mechanisms underlying the hSC-CM automaticity, i.e. the joint role of I_f_ and I_NCX_ in sustaining the hSC-CM spontaneous electrical activity.

The Koivumäki2018 model was mainly based as well on the Ma2011 dataset, but it included also new in-house measurements of I_CaL_ (Koivumäki et al., 2018). Many currents (e.g. I_to_, I_Kr_, I_Ks_, I_K1_) in Koivumäki2018 shared the same structure (with conveniently tuned parameters) observed in the Paci models. A core difference with respect to the Paci models, is a more detailed description of the cell topology. Without need of partial differential equations, Koivumäki2018 can accurately simulate the spatial diffusion of Ca^2+^ in the cytosol, showing good agreement with the *in vitro* data. The formulation was inherited from a previous model of cardiomyocyte derived from mouse embryonic stem cells (Korhonen et al., 2008). However, such refined description of Ca^2+^ diffusion comes at the cost of 100 times longer simulation time.

The Kernik2019 model (Kernik et al., 2019) adopts the same “simple” structure of the Paci models, while integrating a plethora of additional new *in vitro* data (Bellin et al., 2013; Cordeiro et al., 2013; Ma et al., 2015; Es-Salah-Lamoureux et al., 2016; Herron et al., 2016; Veerman et al., 2016; Li et al., 2017; Garg et al., 2018), in addition to the well-known Ma2011 dataset; see Table 1 in the original Kernik et al. publication. However, the authors reformulated seven key ion currents (I_Na_, I_CaL_, I_Kr_, I_Ks_, I_K1_, I_to_ and I_f_). Their motivation was to compensate the lack of time constant information in several published datasets of hSC-CM voltage-clamp data: remarkably, they fit the normalized current recordings to single exponential functions in order to estimate activation/inactivation time constants, to be used then to optimize model parameters. The remaining ion currents followed canonical formulations from literature, e.g. (Shannon et al., 2004; ten Tusscher et al., 2004). Given the shared model structure, Kernik2019 and Paci2020 have similar computing time.

At the time of this paper, two more hSC-like CM models were published, based on reparameterization of hV-CMs models: Zhang et al. (Zhang et al., 2012) and Lemoine et al. (Lemoine et al., 2018). However, their inclusion was out of the scope of this study.

### Drug Responses *in vitro* and *in silico*

The three models simulated consistently drug-induced changes in CTD_90_, all showing CaT prolongation or shortening for the same drug. Although qualitatively in agreement with the *in vitro* experiments, all three models showed smaller percentage variations.

The models demonstrated different susceptibility to generating repolarization abnormalities, summarized in Figure 7, Supplementary Figures S5–S7 and Supplementary Table S2. Paci2020 showed the lowest number of arrhythmic events: the repolarization failure only for the highest concentration of I_K1_ blocker BaCl_2_. In case of drugs blocking I_Kr_, this model consistently prolonged the duration of the CaT, but no EAD or DAD appeared. Kernik2019 failed to repolarize in response to six drugs (for at least one formulation in case of multiple drug formulations) blocking prevalently I_Kr_, although it never developed EADs or DADs. Finally, Koivumäki2018 showed the highest variety of arrhythmic events, including EADs, DADs, repolarization failure and irregular rhythm for nine drugs. The drugs that triggered arrhythmic events in Koivumäki2018 and Kernik2019 were all classified as “Known Risk of TdP” by CredibleMeds (Woosley et al., 2020), except for Ranolazine (“Conditional Risk of TdP”), BaCl_2_ and Nimodipine (not reviewed) and Verapamil (not classified).

Automaticity is one key-feature of hSC-CMs, being a macromarker of the successful differentiation into CMs, but also a sign of hSC-CM immaturity when compared to adult cardiac cells. Automaticity in Paci2020 and Koivumäki2018 stopped (or produced minor residual electrical activity) for the drugs strongly blocking I_Na_ and/or I_CaL_ (e.g. bepridil, ranolazine I, flecainide, lidocaine, mexiletine, nimodipine, nisoldipine and phenytoin). Conversely, only bepridil and nisoldipine, Ca^2+^ current blockers, terminated the spontaneous electrical activity in Kernik2019: this model exhibited the stronger automaticity, as we report in our sensitivity analysis (Figure 6).

It must be noted that the *in vitro* data included observations of six recordings, and only a few of them showed arrhythmic events or quiescence following drug administration, thus highlighting variability in the *in vitro* responses. This is not different from the behavior of the three *in silico* models.

Since *in silico* drug tests are based on simulations, not only the biophysical accuracy of the models, but also the precision of the characterization of drugs is critical (in terms of what ion channels are affected and to what extent). Our tests showed that the effect of a certain drug concentration can be very different depending on the *in vitro* characterization, e.g. moxifloxacin I and II vs moxifloxacin III. Recently, Zhou et al. (Zhou et al., 2020) demonstrated how reducing the amount of ion currents considered in drug characterizations (e.g. I_Na_, I_Kr_ and I_CaL_ vs I_Kr_ only) reduces the specificity of the *in silico* predictions, marking safe drugs as at risk. The same authors examined also how moderate changes in IC_50_ and variable Hill coefficients (representing the steepness of the drug dose-response curves) lead to divergent predictions using the same population of hV-CM models, e.g. for sotalol and verapamil.

### Accounting for Variability

High phenotypical and electrophysiological variability is one key feature of hSC-CMs and one of the reason of skepticism (together with their structural immaturity (Knollmann, 2013; Koivumäki et al., 2018)) when using them in real-world applications, where results should be then translated to the human. However, it is important to acknowledge that, despite the limitations, *in vitro* drug tests on hSC-CMs showed already their capability to meet and surpass traditional preclinical tests on animal models, considering as metric the CaT prolongation (Pfeiffer et al., 2016).

A recent work by Biendarra-Tiegs et al. (Biendarra-Tiegs et al., 2019) identifies many potential sources of variability in producing induced pluripotent stem cells (abnormalities at genetic/epigenetic level, differences among donors, endogenous signaling and extrinsic factors such as culture conditions, media, substrate, pH) and differentiating them into cardiomyocytes (differentiation methods, maturation protocols and level of maturation of hSC-CMs when *in vitro* measurements are done). By deep-diving in the eleven *in vitro* datasets used to build the three *in silico* models here studied, we observe already many potential sources of variability:

• Four datasets used only iCell pluripotent stem cells (including the Ma2011 dataset, used for all the three models), six datasets used only in-house cells from donors, in most cases healthy, but e.g. Bellin et al. report fibroblasts from a LQT2 patient (hSC-CMs were corrected later) (Bellin et al., 2013). For the dataset from (Ma et al., 2015), the authors used both iCell hSCs (for control) and in-house hSC-CMs produced from a LQT1 patient.
• Combinations of different reprogramming factors were used to induce the pluripotency in the in-house cells from donors, e.g. combinations from (Takahashi et al., 2007; Yu et al., 2007).
• Different methods were used to differentiate hSCs into CMs: embryoid bodies (e.g. (Garg et al., 2018)), coculturing with mouse visceral endodermal-like cells (END-2) (e.g. in-house cells for the Paci2020 model), modulation of the WNT signaling (e.g. in-house cells for I_CaL_ measures for the Koivumäki2018 model and (Ma et al., 2015)).

There is clear need for standardization for *in vitro* hSC-CM drug assays. For example, Blinova et al. (Blinova et al., 2018) demonstrated that, despite hSC-CM variability, good consensus can be achieved across multiple laboratories on drug test results, by means of a scrupulously planned multi-site study. In addition to the differences in the equations and structures of the Paci2020, Koivumäki2018 and Kernik2019 models, the aforementioned sources of variability identified in the *in vitro* datasets could play a role in the differences in the APs and CaTs simulated by the three *in silico* models, also in basal conditions.

Nevertheless, it is not common considering sources of variability such as differences among donors or maturation level when modeling the electrophysiology of hSC-CMs. What has been already done with the three *in silico* models we considered is to account for the *in vitro* variability observed in AP features, responses to drugs, and sensitivity to channelopathies by developing *in silico* hSC-CM populations, following different approaches. In (Paci et al., 2020), Paci et al. randomly sampled 22 models parameters (maximum conductances and dynamic parameters, covering all the main ion currents/exchangers/pumps in the model) using the Latin Hypercube sampling, thus obtaining a random population. The random population was then experimentally calibrated using AP and CaT features. Kernik et al. followed a different approach, sampling the model parameters (five dynamic parameters and maximum conductance for five ion currents) from independent distributions, each one centered on the baseline value of each of the chosen parameters. Also, Koivumäki2018 was used as baseline for a population where seven maximum conductances/currents were sampled according to literature data, but only two AP features (maximum diastolic potential and AP peak) were used as exclusion criteria.

## Conclusion

In this paper, we investigate the simulated response of three hSC-CM models to drug action in order to 1) highlight the differences in their principal functional features, and 2) compare their drug responses, based on a panel of *in vitro* data. We extract the following recommendations.

• In order to assess the potential arrhythmogenic activity of a compound on a single simulation, Koivumäki2018 might be the best option because of its high sensitivity to I_Na_, I_CaL_ and I_Kr_ blocks. However, Koivumäki2018 could predict an excess of false positives. Also, for a population-based study, the Koivumäki2018 model requires an excessively long running time.
• To account for hSC-CM variability (in terms of responses to drugs and sensitivity to channelopathies), the Paci2020 and Kernik2019 models are more suitable to be baselines for populations of *in silico* models. Furthermore, because of the very similar structure and running time of Paci2020 and Kernik2019, variability could also be simulated in population-based studies not only by sampling parameters (according to the techniques presented in this section), but also by using both models as baselines for a hybrid population.

Our simulation study shows differences in the drug responses across the different hSC-CM models, which could reflect the variability in the *in vitro* data used to design them and, more generally, of the results of *in vitro* drug tests done on hSC-CMs.

## Data Availability

The raw data supporting the conclusions of this article will be made available on request by contacting the authors, without undue reservation.
