# Supplementary material for: Comparison of the Simulated Response of Three in Silico Human Stem Cell-Derived Cardiomyocytes Models and in Vitro Data Under 15 Drug Actions
Source: Front Pharmacol. 2021 Mar 15;12:604713. doi: 10.3389/fphar.2021.604713 (PMC8033762; doi:10.3389/fphar.2021.604713)
Supplement: Supplementary file 1 [file datasheet1.pdf]

Supplementay Material

Comparison of the Simulated Response of Three *in Silico* Human Stem Cell-Derived Cardiomyocytes Models and *in Vitro* Data Under 15 Drug Actions

Michelangelo Paci*, Jussi T. Koivumäki, Hua Rong Lu, David J. Gallacher, Elisa Passini, Blanca Rodriguez

*** Correspondence:** Michelangelo Paci: michelangelo.paci@tuni.fi

1 Supplementary Figures

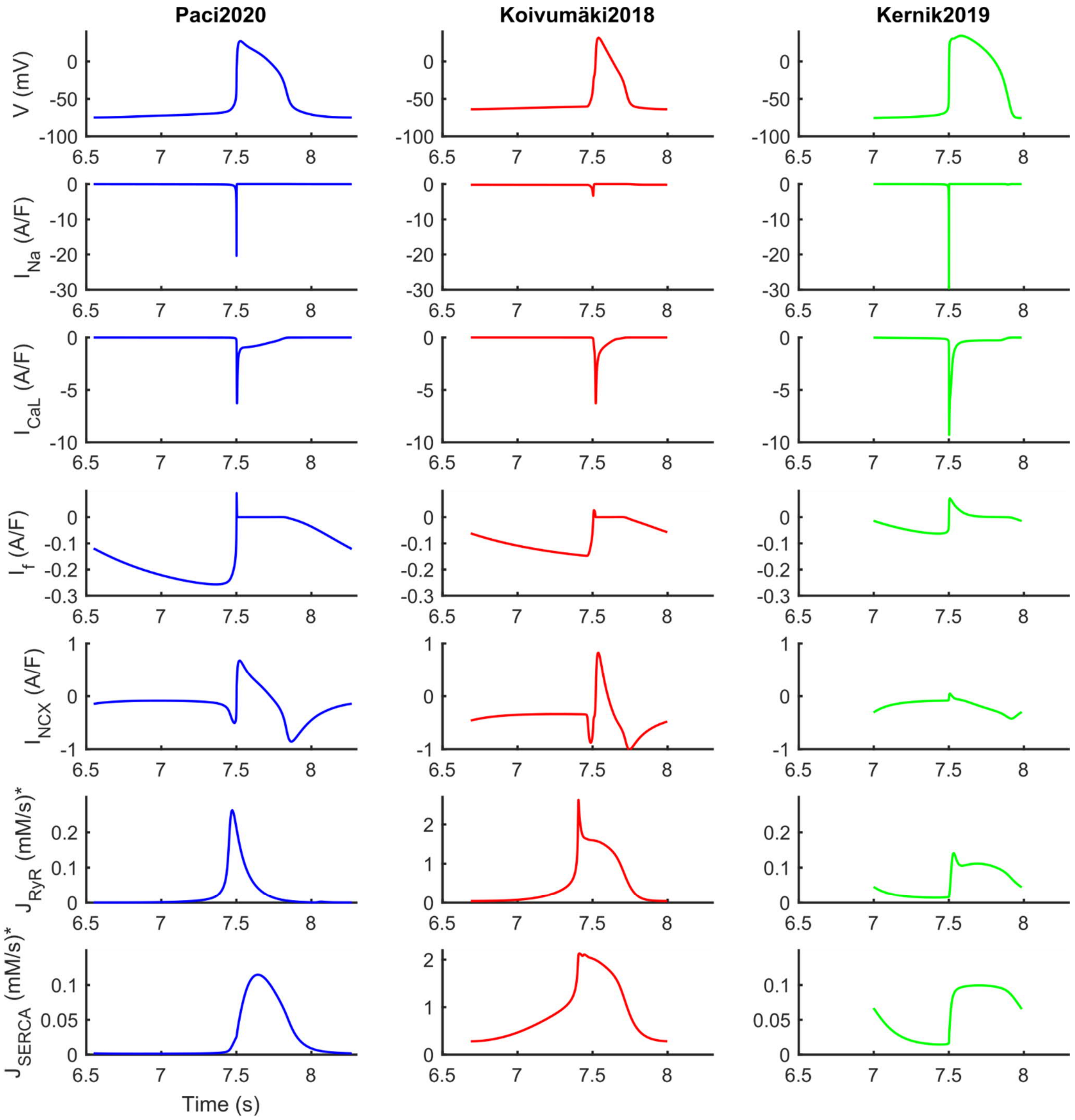

Figure S1. Expanded version of Figure 1 in the main manuscript. Ion currents underlying the steady-state action potentials in the Paci2020 (blue), Koivumäki2018 (red) and Kernik2019 (green) models: membrane potential (V), fast Na^+ current (I_{Na}), L-type Ca^{2+} current (I_{CaL}), Funny current (I_f), Na^+-Ca^{2+} exchanger (I_{NCX}), release current from sarcoplasmic reticulum (J_{RyR}) and SERCA pump (J_{SERCA}). The y scale of the ion current/fluxes marked with a star was adapted to each model to improve visibility.

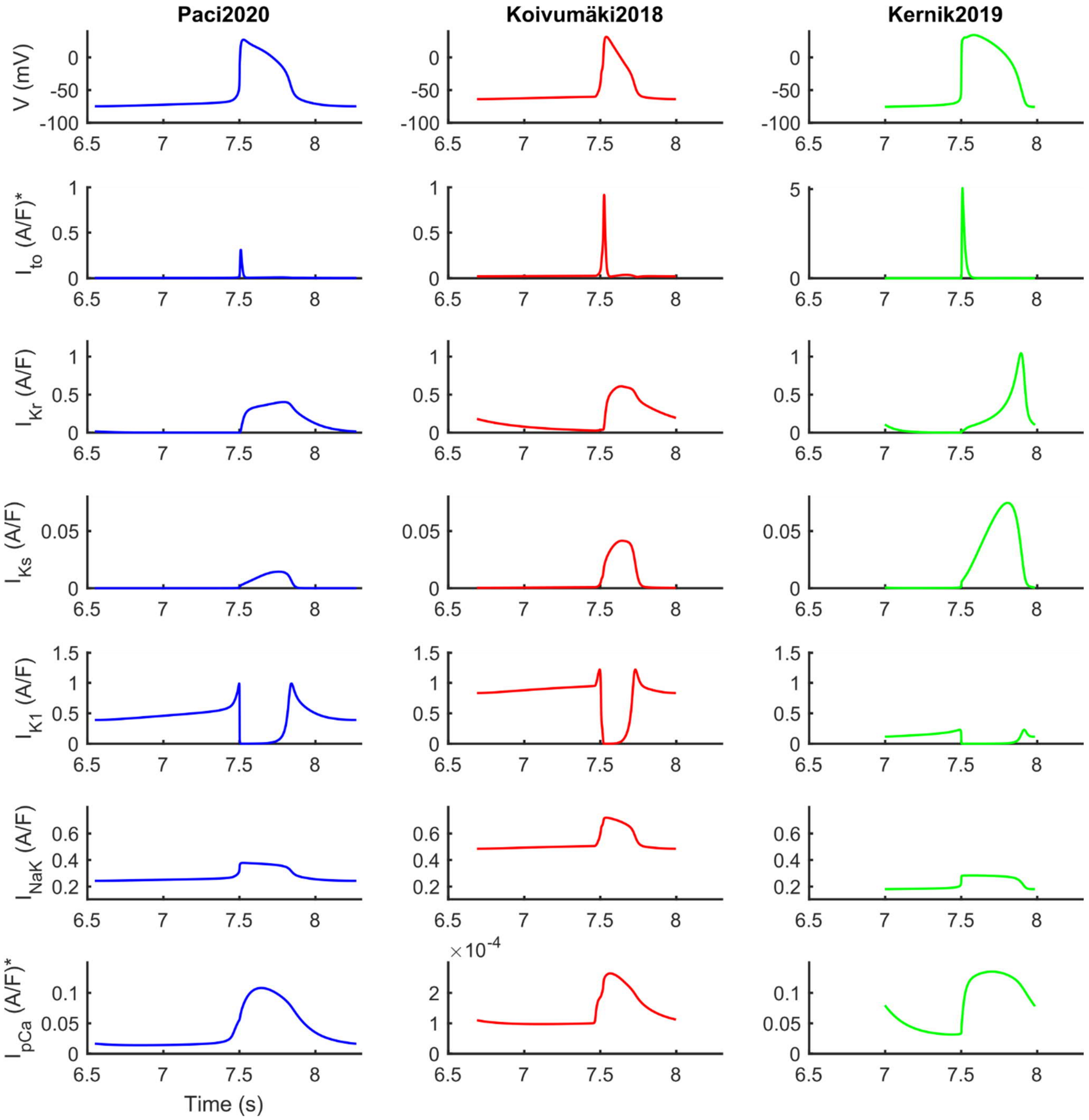

Figure S2. Expanded version of Figure 1 in the main manuscript. Ion currents underlying the steady-state action potentials in the Paci2020 (blue), Koivumäki2018 (red) and Kernik2019 (green) models: membrane potential (V), transient outward K^+ current (I_{to}), rapid delayed rectifying K^+ current (I_{Kr}), slow delayed rectifying K^+ current (I_{Ks}), inward rectifying K^+ current (I_{K1}), Na^+-K^+ pump (I_{NaK}) and sarcolemma Ca^{2+} pump (I_{pCa}). The y scale of the ion current/fluxes marked with a star was adapted to each model to improve visibility.

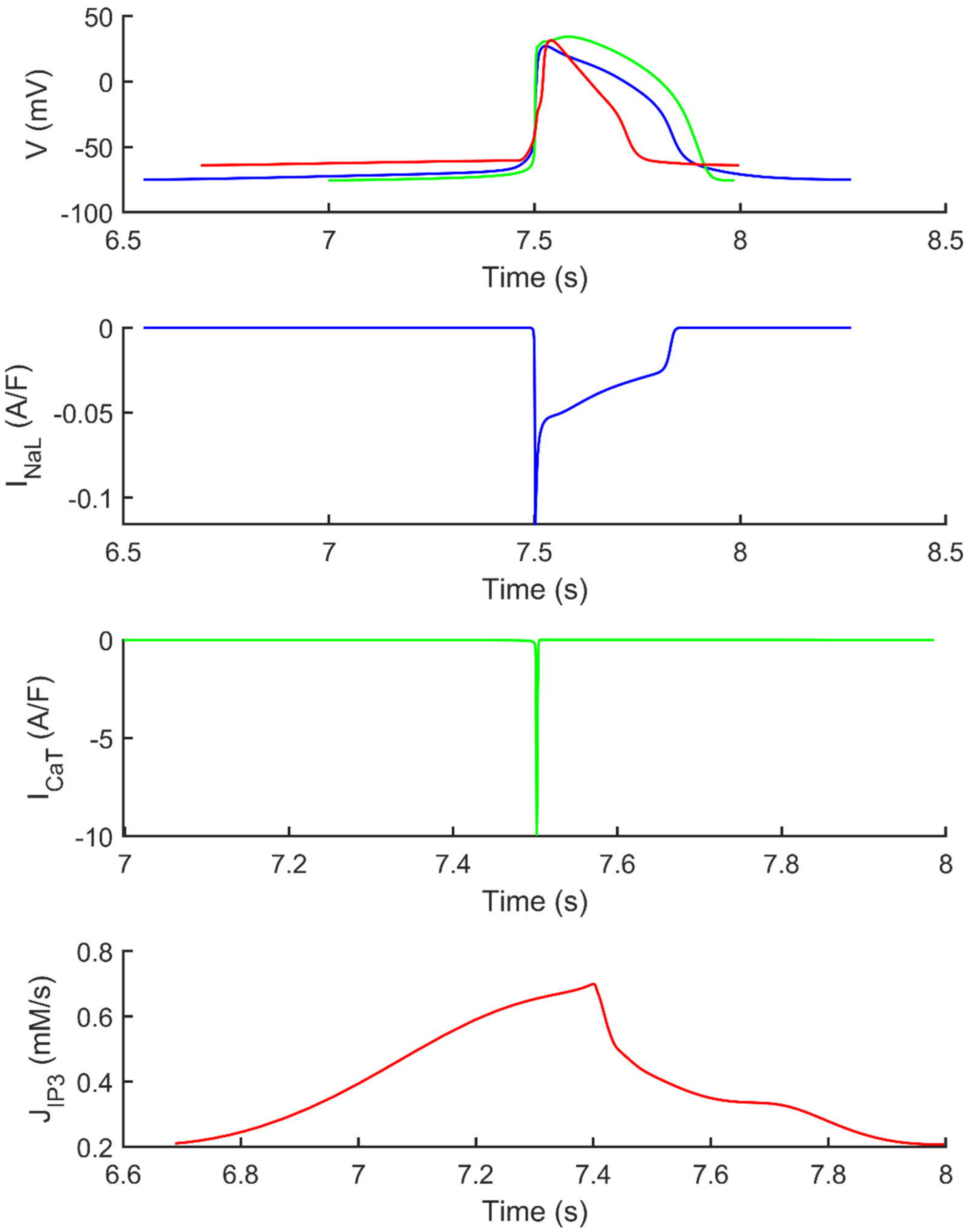

Figure S3. Ion currents/fluxes included only in one of the three models. Paci2020 (blue), Kernik2019 (green) and Koivumäki2018 (red). From top to bottom: membrane potential, Late Na^+ current (I_{NaL}), T-type Ca^{2+} current (I_{CaT}), and Inositol 1,4,5 triphosphate receptor-mediated Ca^{2+} release (J_{IP3}).

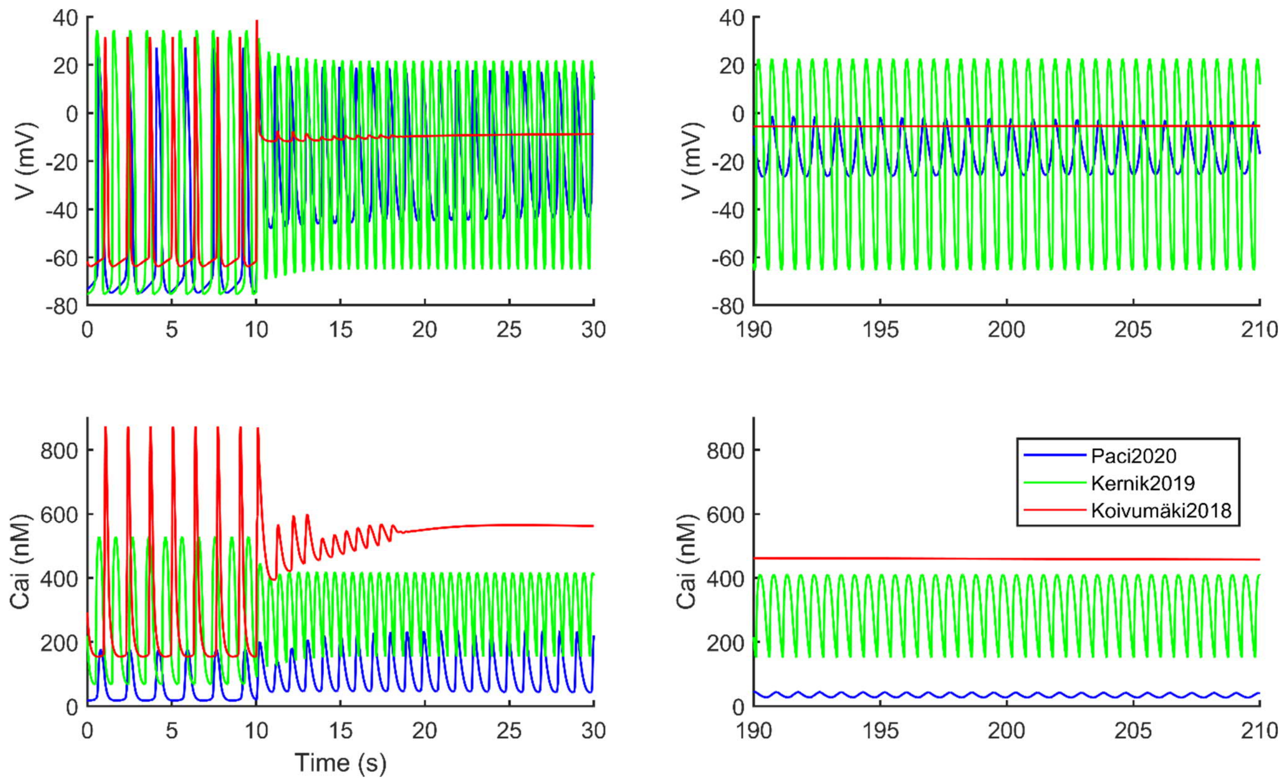

Figure S4. Effect of 100 µM $BaCl_2$ (concentration C3) on the three *in silico* models.

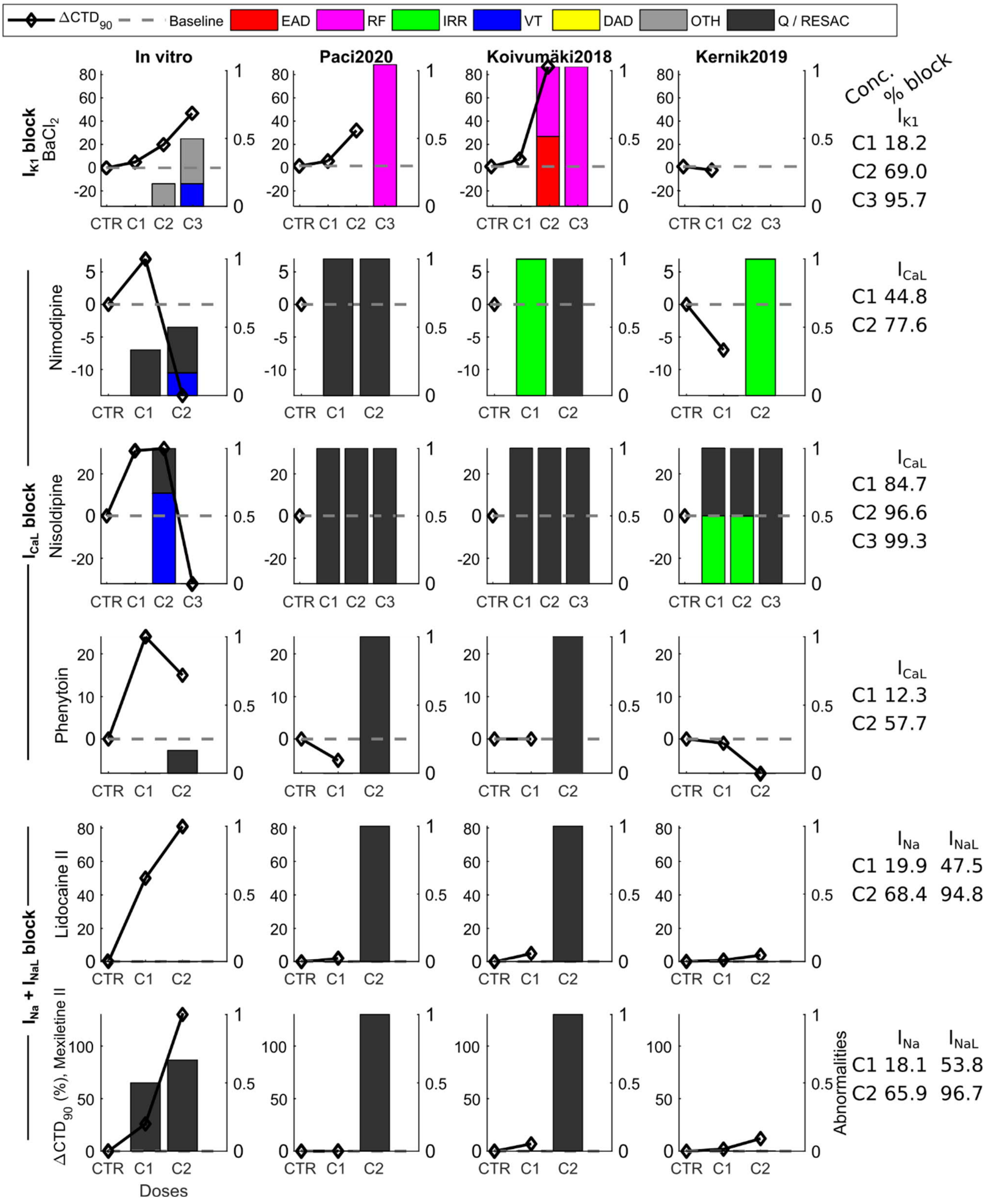

Figure S5. Left axis: percentage change in Ca^{2+} transient (CaT) duration with respect to baseline as consequence of drug administration (ΔCTD_{90}%). *In vitro* and *in silico* experiments are reported as black diamonds. In case of missing *in silico* data points, the simulations produced AP/CaT arrhythmic events and it was not possible computing the CTD_{90}. $BaCl_2$ in Kernik2019 slightly depolarized the

maximum diastolic potential and induced high rate Ca^{2+} oscillations, but did not trigger RF. Right axis: occurrence of arrhythmic events or cessation of spontaneous activity. *In vitro* data are reported as percent of events recorded on the six observations. In case of multiple *in silico* events, the bar was split accordingly. Early afterdepolarization (EAD), repolarization failure (RF), irregular rhythm (IRR), ventricular tachycardia (VT), delayed afterdepolarization (DAD), other events observed *in vitro* (OTH), cessation of spontaneous activity and/or residual electrical activity (Q / RESAC). CTD_{90} values for Koivumäki2018 for $BaCl_2$ concentration C2 were manually computed in presence of EAD-like abnormalities on the CaTs.

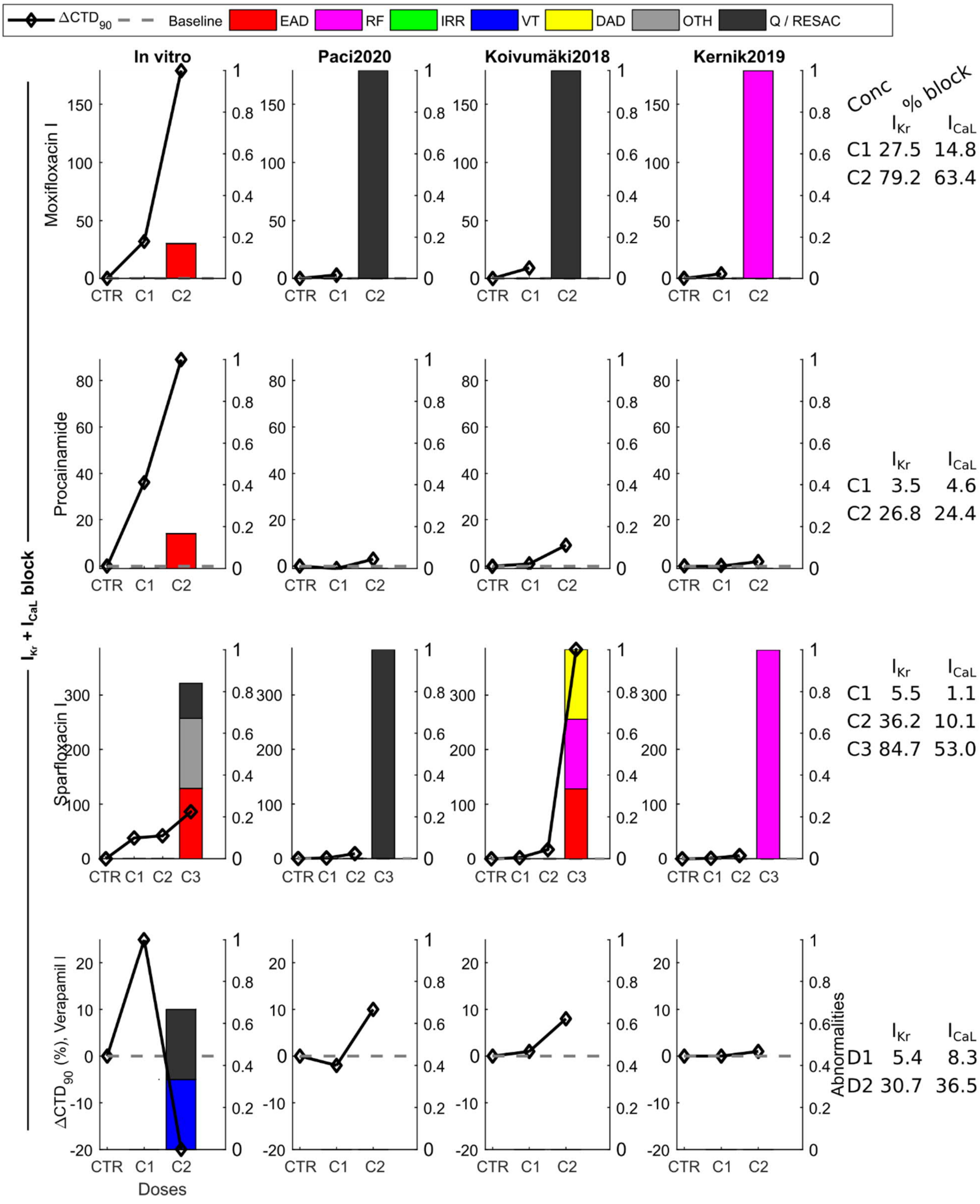

Figure S6. Left axis: percentage change in Ca^{2+} transient (CaT) duration with respect to baseline as consequence of drug administration (ΔCTD_{90}%). *In vitro* and *in silico* experiments are reported as black diamonds. In case of missing *in silico* data points, the simulations produced AP/CaT arrhythmic events and it was not possible to compute the CTD_{90}. Right axis: occurrence of arrhythmic events or cessation of spontaneous activity. *In vitro* data are reported as percent of events recorded on the six

observations. In case of multiple *in silico* events, the bar was split accordingly. Events were classified as in Figure 3. CTD_{90} values for Koivumäki2018 for sparfloxacin concentration C3 were manually computed in presence of EAD-like abnormalities on the CaTs.

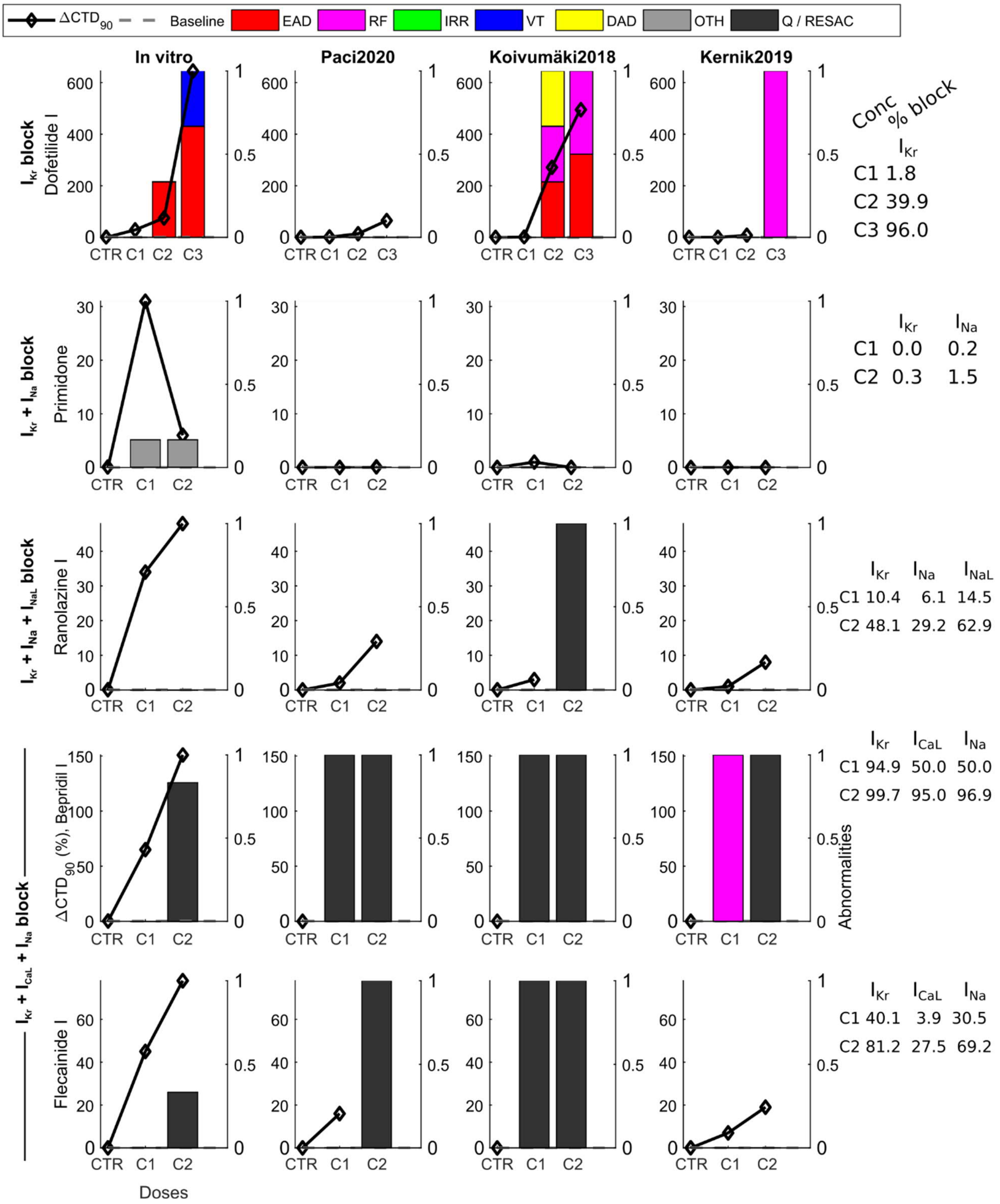

Figure S7. Left axis: percentage change in Ca^{2+} transient (CaT) duration with respect to baseline as consequence of drug administration (ΔCTD_{90}%). *In vitro* and *in silico* experiments are reported as black diamonds. In case of missing *in silico* data points, the simulations produced AP/CaT arrhythmic events and it was not possible computing the CTD_{90}. Right axis: occurrence of arrhythmic events or cessation of spontaneous activity. *In vitro* data are reported as percent of events recorded on the six

observations. In case of multiple *in silico* events, the bar was split accordingly. Events were defined as in Figure 3. CTD_{90} values for Koivumäki2018 for dofetilide concentration C2 and C3 were manually computed in presence of EAD-like abnormalities on the CaTs.

2 Supplementary Tables

Table S1: IC_{50} and Hill's coefficients (in brackets) for the 15 drugs tested *in vitro*. If a drug has been characterized by multiple (IC_{50}, Hill's coefficient) sets, the name of the drug is followed by I, II and III. This table was adapted from (Passini et al., 2017) and the drug names were kept consistent with it.

Compound List		IC_{50}(h), with IC_{50} in µM						
		I_{Na}	I_{Kr}	I_{CaL}	I_{NaL}	I_{Ks}	I_{to}	I_{K1}
**1**	**$BaCl_2$**		257(1)					4.5(1)
**2**	**Bepridil I**	1(1.49)	0.09(1.21)	1(1.28)				
**3**	**Bepridil II**	2.3(1.26)	0.16(0.88)	1(1.28)				
**4**	**Bepridil III**	2.929(1.2)	0.149(0.9)	2.808(0.6)	1.814(1.4)			
**5**	**Dofetilide I**	31.9(0.54)	0.013(1.56)	201(1)		135(1)	300(1)	
**6**	**Dofetilide II**	162.1(1)	0.03(1.2)	26.7(1)				
**7**	**Dofetilide III**		0.001(0.6)					
**8**	**Flecainide I**	3.19(0.71)	1.64(0.81)	27.1(0.97)				
**9**	**Flecainide II**	6.2(1.14)	1.5(0.88)	27.1(0.97)				
**10**	**Flecainide III**	6.677(1.9)	0.692(0.8)	25.599(1.4)	18.87(0.6)		9.266(0.7)	
**11**	**Lidocaine II**	44(0.94)	300(1)		10.79(1.3)			
**12**	**Mexiletine II**	49.7(0.94)	69.4(1.11)	203(0.75)	8.957(1.4)	32.6(0.92)	367(0.91)	
**13**	**Moxifloxacin I**	1563(1)	79(1)	173(1)				
**14**	**Moxifloxacin II**	1112(1)	86.2(0.94)	173(1)				
**15**	**Moxifloxacin III**		93.041(0.6)		382.337(1.1)	50.321(1)		
**16**	**Nimodipine**		45.6(1)	0.139(0.63)				
**17**	**Nisoldipine**	45(0.72)	49.3(0.84)	0.009(0.71)	61.7(0.71)	52.8(1.03)		
**18**	**Phenytoin**	72.4(1.06)	147(1)	21.9(0.99)				
**19**	**Primidone**	640(1)	3360(1)					
**20**	**Procainamide**	746.6(1)	272.4(1)	389.5(0.83)				
**21**	**Ranolazine I**	30.2(0.8)	10.9(0.9)	172(0.6)	5.9(1)			
**22**	**Ranolazine II**		6.49(0.8)		7.887(0.9)			
**23**	**Sparfloxacin I**	1465(1)	17.7(0.99)	88.8(1)				
**24**	**Sparfloxacin II**	2555(1)	22.1(0.93)	88.8(1)				
**25**	**Verapamil I**	32.5(1.33)	0.25(0.89)	0.2(0.8)				
**26**	**Verapamil II**	7.2(0.95)	0.83(1.17)	0.1(0.7)	6.1(1.24)	65.6(0.92)		9.03(1)
**27**	**Verapamil III**		0.499(1.1)	0.202(1.1)				

Table S2. Percent CaT duration variation with respect to baseline (ΔCTD_{90}%), arrhythmic events (ARR) and automaticity cessation (AC) *in vitro* and *in silico*. Arrhythmic events: early and delayed after-depolarization (EAD, DAD), repolarization failure (RF), irregular rhythm (IRR), ventricular tachycardia-like rhythm (VT) and other arrhythmic events (OTH). Automaticity cessation: quiescence (Q) and residual activity (RESAC). *: *in silico* ΔCTD_{90}% marked with a star were computed on EADs. **: depolarized maximum diastolic potential, high rate Ca^{2+} oscillations but no RF.

		In vitro		Paci2020		Koivumäki2018		Kernik2019	
Drug	Conc. (µM)	ΔCTD_{90} %	ARR, AC	ΔCTD_{90} %	ARR, AC	ΔCTD_{90} %	ARR, AC	ΔCTD_{90} %	ARR, AC
$BaCl_2$	1	5	---	4	---	6	---	-3	---
$BaCl_2$	10	20	OTH 1/6	29	---	83*	EAD RF	**	---
$BaCl_2$	100	47	VT 1/6 OTH 2/6	---	RF	---	RF	**	---
Bepridil I	1	65	---	---	Q	---	RESAC	---	RF
Bepridil I	10	151	Q 5/6	---	Q	---	RESAC	---	Q
Bepridil II	1	65	---	---	Q	---	RESAC	---	RF
Bepridil II	10	151	Q 5/6	---	Q	---	RESAC	---	Q
Bepridil III	1	65	---	25	---	---	RESAC	---	RF
Bepridil III	10	151	Q 5/6	---	Q	---	RESAC	---	Q
Dofetilide I	0.001	29	---	1	---	1	---	0	---
Dofetilide I	0.01	75	EAD 2/6	14	---	277*	EAD DAD RF	8	---
Dofetilide I	0.1	645	EAD 4/6 VT 4/6	65	---	503*	EAD RF	---	RF
Dofetilide II	0.001	29	---	1	---	0	---	0	---
Dofetilide II	0.01	75	EAD 2/6	7	---	8	---	4	---
Dofetilide II	0.1	645	EAD 4/6 VT 4/6	35	---	610*	EAD DAD RF	---	RF
Dofetilide III	0.001	29	---	17	---	881*	EAD DAD RF	12	---
Dofetilide III	0.01	75	EAD 2/6	35	---	523*	EAD DAD RF	---	RF
Dofetilide III	0.1	645	EAD 4/6 VT 4/6	62	---	641*	EAD DAD RF	---	RF
Flecainide I	1	45	---	16	---	---	RESAC	7	---
Flecainide I	10	78	Q 2/6	---	Q	---	RESAC	19	---

		In vitro		Paci2020		Koivumäki2018		Kernik2019	
Drug	Conc. (μM)	ΔCTD_{90} %	ARR, ABN	ΔCTD_{90} %	ARR, ABN	ΔCTD_{90}	ARR, ABN	ΔCTD_{90} %	ARR, ABN
Flecainide II	1	45	---	14	---	219*	EAD DAD RF	8	---
Flecainide II	10	78	Q 2/6	---	Q	---	RESAC	---	RF
Flecainide III	1	45	---	20	---	599*	EAD DAD RF	---	RF
Flecainide III	10	78	Q 2/6	---	Q		RESAC	---	RF
Lidocaine II	10	50	---	2	---	5	---	1	---
Lidocaine II	100	81	---	---	Q	---	RESAC	4	---
Mexiletine II	10	26	Q 3/6	0	---	7	---	2	---
Mexiletine II	100	130	Q 4/6	---	Q	---	RESAC	12	---
Moxifloxacin I	30	32	---	3	---	9	---	4	---
Moxifloxacin I	300	179	EAD 1/6	---	Q	---	RESAC	---	RF
Moxifloxacin II	30	32	---	3	---	9	---	4	---
Moxifloxacin II	300	179	EAD 1/6	---	Q	---	RESAC	---	RF
Moxifloxacin III	30	32	---	12	---	141*	EAD DAD RF	8	---
Moxifloxacin III	300	179	EAD 1/6	30	---	---	RF	---	RF
Nimodipine	0.1	7	Q2/6	---	Q	---	IRR	-7	---
Nimodipine	1	-14	VT 1/6 Q 2/6	---	Q	---	RESAC	---	IRR
Nisoldipine	0.1	31	---	---	Q	---	RESAC	---	IRR Q
Nisoldipine	1	32	VT 4/6 Q 2/6	---	Q	---	RESAC	---	IRR Q
Nisoldipine	10	-32	---	---	Q	---	RESAC	---	Q
Phenytoin	3	24	---	-5	---	0	---	-1	---
Phenytoin	30	15	Q 1/6	---	Q	---	RESAC	-8	---
Primidone	1	31	OTH 1/6	0	---	1	---	0	---
Primidone	10	6	OTH 1/6	0	---	0	---	0	---
Procainamide	10	36	---	-1	---	1	---	0	---
Procainamide	100	89	EAD 1/6	-3	---	9	---	2	---
Ranolazine I	1	34	---	2	---	3	---	1	---
Ranolazine I	10	48	---	14	---	---	RESAC	8	---
Ranolazine II	1	34	---	6	---	7	---	3	---
Ranolazine II	10	48	---	20	---	591*	EAD DAD RF	---	RF

		In vitro		Paci2020		Koivumäki2018		Kernik2019	
Drug	Conc. (μM)	ΔCTD_{90} %	ARR, ABN	ΔCTD_{90} %	ARR, ABN	ΔCTD_{90}	ARR, ABN	ΔCTD_{90} %	ARR, ABN
Sparfloxacin I	1	38	---	1	---	2	---	1	---
Sparfloxacin I	10	42	---	9	---	17	---	6	---
Sparfloxacin I	100	86	EAD 2/6<br>Q 1/6<br>OTH 2/6	---	Q	386*	EAD<br>DAD<br>RF	---	RF
Sparfloxacin II	1	38	---	1	---	2	---	1	---
Sparfloxacin II	10	42	---	8	---	13	---	5	---
Sparfloxacin II	100	86	EAD 2/6<br>Q 1/6<br>OTH 2/6	---	Q	324*	EAD<br>DAD<br>RF	---	RF
Verapamil I	0.01	25	---	-2	---	1	---	0	---
Verapamil I	0.1	-20	VT 2/6<br>Q 2/6	---	Q	8	---	1	---
Verapamil II	0.01	25	---	-9	---	-1	---	-1	---
Verapamil II	0.1	-20	VT 2/6<br>Q 2/6	---	Q	---	IRR	-7	---
Verapamil III	0.01	25	---	-1	---	0	---	0	---
Verapamil III	0.1	-20	VT 2/6<br>Q 2/6	---	Q	2	---	-2	---

References

Passini, E., Britton, O. J., Lu, H. R., Rohrbacher, J., Hermans, A. N., Gallacher, D. J., et al. (2017). Human In Silico Drug Trials Demonstrate Higher Accuracy than Animal Models in Predicting Clinical Pro-Arrhythmic Cardiotoxicity. *Front. Physiol.* 8, 1–15. doi:10.3389/fphys.2017.00668.
